# Adaptation of the *Spalax galili* transcriptome to hypoxia may underlie the complex phenotype featuring longevity and cancer resistance

**DOI:** 10.1038/s41514-025-00206-3

**Published:** 2025-03-06

**Authors:** Gesa Poetzsch, Luca Jelacic, Leon Dammer, Sören Lukas Hellmann, Michelle Balling, Miguel Andrade-Navarro, Aaron Avivi, Imad Shams, Anne Bicker, Thomas Hankeln

**Affiliations:** 1 Molecular Genetics & Genome Analysis, Institute of Organismic and Molecular Evolution, Faculty of Biology, Johannes Gutenberg-University, Mainz, Germany; 2 Computational Biology and Data Mining Group, Institute of Organismic and Molecular Evolution, Johannes Gutenberg University, Mainz, Germany; 3 Institute of Evolution, University of Haifa, Mount Carmel, Haifa Israel; 4 Department of Evolutionary and Environmental Biology, Faculty of Natural Sciences, University of Haifa, Mount Carmel, Haifa Israel; 5 Present Address: Nucleic Acids Core Facility, Faculty of Biology, Johannes Gutenberg-University, Mainz, Germany; 6 Present Address: Department of Medicine I, Johannes Gutenberg University Medical Center, Mainz, Germany

**Keywords:** Molecular biology, Transcription

## Abstract

In the subterranean rodent *(Nanno)spalax galili*, evolutionary adaptation to hypoxia is correlated with longevity and tumor resistance. Adapted gene-regulatory networks of *Spalax* might pinpoint strategies to maintain health in humans. Comparing liver, kidney and spleen transcriptome data from *Spalax* and rat at hypoxia and normoxia, we identified differentially expressed gene pathways common to multiple organs in both species. Body-wide interspecies differences affected processes like cell death, antioxidant defense, DNA repair, energy metabolism, immune response and angiogenesis, which may play a crucial role in *Spalax*’s adaptation to environmental hypoxia. In all organs, transcription of genes for genome stability maintenance and DNA repair was elevated in *Spalax* versus rat, accompanied by lower expression of aerobic energy metabolism and proinflammatory genes. These transcriptomic changes might account for the extraordinary lifespan of *Spalax* and its cancer resistance. The identified gene networks present candidates for further investigating the molecular basis underlying the complex *Spalax* phenotype.

## Introduction

Understanding of principles of ageing and an extension of lifespan is a key interest of biomedical research^1,2^. Organisms with an extended lifespan might increase our insight into biological mechanisms of (healthy) ageing^3^. Rodents showing unusual longevity have consequently become valuable models for studying molecular mechanisms of geroprotection^4^. One of those emerging model taxa is *Nannospalax galili* (also called *Spalax* or *blind mole rat, BMR*), a member of the superspecies *Spalax ehrenbergi* from the muroid family of *Spalacidae*, a group of fossorial rodents that diverged from the phylogenetic lineages leading to mouse and rat approximately 47 million years ago^5,6^. While laboratory rats have an average lifespan of 3 years^7^, *Spalax* reaches up to 21 years without displaying clear signs of ageing or age-related disorders^8^. Moreover, in 50 years of *Spalax* research, there has never been a reported case of spontaneous tumor formation among thousands of observed individuals. Even exposition to potent carcinogens failed—with very rare exceptions—to induce tumor growth in *Spalax*, whereas 100% of exposed mice and rats in the same study developed cancer^8^. Interestingly, the blind mole rat shares these important traits with another fossorial rodent, the African naked mole rat (NMR) *Heterocephalus glaber*^9,10^, which is adapted to moderate, chronic hypoxia in its populated, eusocial underground colonies and which reaches lifespans of up to 30 years and develops cancer only in rare cases^9^. Since *Spalax* and *H. glaber* belong to different clades of the rodent phylogeny, they probably evolved these traits convergently during speciation in adaptation to hypoxia^5,11^. Hence, these fossorial rodents, which efficiently cope with the multiple stresses of underground life, are now studied to reveal the adaptive processes involved in tissue oxygenation, tumor formation and longevity^9,12,13^.

*Spalax* individuals inhabit self-dug burrows to avoid predators and spend most of their lifes solitarily^14^. Due to flooding of its burrow system during seasonal rainfalls, *N. galili* overcomes the most severe fluctuations in oxygen and carbon dioxide among all *S. ehrenbergi* subspecies. O_2_ concentrations of only 7% were measured in flooded *Spalax* burrows^15^. Under laboratory conditions, *N. galili* survived 14 h at 3% O_2_, whereas rats died after 2-4 h^16^. This efficient hypoxia tolerance is realized in *Spalax* by a combination of physiological adaptations. As a shared feature of many subterranean rodents, *Spalax* displays a very low basal metabolic rate even under normoxic conditions, which limits oxygen consumption^14^. In addition, *Spalax* possesses a significantly smaller total skeletal muscle mass than comparatively sized rodents^17^ Oxygen distribution, on the other hand, is increased by elevated capillary and mitochondrial density^18^. In contrast to *S. ehrenbergi* subspecies living in arid, well-oxygenated and permeable soil, *N. galili* shows elevated concentrations of red blood cells and hemoglobin^19^. Together, these features are thought to guarantee unimpaired body functions even under challenging atmospheric oxygen conditions. However, it is suspected that many more adaptations are found on the molecular level which are hitherto incompletely understood.

It has been hypothesized that the three combined phenotypes - hypoxia tolerance, longevity, tumor resistance - could be mechanistically linked at the genetic level^20–24^. Comparative genomics and molecular analyses have provided evidence that positively selected sequence changes in coding genes, but also changes in gene regulation have contributed to adaptation in the subterranean rodents^5,13,25–27^. In *Spalax*, for example, amino acid replacements in the crucial tumor suppressor p53 render the protein unable to activate some of its apoptosis-regulating target genes^28^, while over-activating targets mediating cell-cycle arrest. This suggests an adaptive strategy to prevent hypoxia-induced cell death, while allowing DNA repair to take place. Pseudogenization associated with visual perception and DNA repair is another evolutive feature of *Spalax* observed at the genomic level^29^. Other gene-focused studies revealed substantially increased mRNA and protein expression of key players involved in a variety of functions in *Spalax* organs in comparison to tissues from the non-hypoxia-adapted rat, e.g., elevated *Hif1a*^30^ and *Epo* levels^16^, overexpression of respiratory globin proteins^31^, ROS defense enzymes^32^, and tissue vascularization genes^33^. Large-scale transcriptome analyses of selected *Spalax* organs uncovered additional gene-regulatory adaptations, e.g., an attenuated transcriptional response of energy metabolism genes under normoxic conditions in brain^26^ and in response to hypoxia in liver tissue^13^. Both, *Spalax* liver and brain transcriptomes (in comparison to rat) revealed constitutively increased expression of important DNA damage repair genes, such as the base excision repair-associated *glycosylase gene Endonuclease VIII-like 2* (*Neil2*), the *Xeroderma pigmentosum-associated* (*Xpa*) nucleotide excision repair gene and the progeria-associated helicase Werner (*Wrn*)^13,26^. In addition, replicative stress response genes interacting with *Wrn*, such as the *Ataxia telangiectasia and Rad3 related gene* (*Atr*), the *Serine/Threonine Kinase Ataxia Telangiectasia Mutated gene* (*Atm*) and members of the *Fanconi-Anemia pathway* showed increased mRNA expression in *Spalax* livers, compared to rat^13^. Altogether, these genetic and gene regulatory changes may ensure genome stability in *Spalax* when severe hypoxia and subsequent reoxygenation challenge tissues and cells, e.g., via production of toxic ROS^26^.

The results above support the idea that gene expression networks in *Spalax*, which e.g. increase genome stability may explain decreased ageing and tumorigenesis as “side effects” of hypoxia adaptation. We hypothesize that such crucial adaptive features are found body-wide, i.e. in the transcriptomes of many vital tissues. To investigate this, we generated and analyzed RNA-Seq data from additional *Spalax* and rat organs (kidney, spleen) obtained from specimen subjected to hypoxia (6% O_2_) and normoxia as controls. Including previous RNA-seq data, we conducted meta-transcriptomic comparisons across *Spalax* tissues to elucidate tissue-overarching versus -specific gene regulatory responses and discuss our results with respect to molecular mechanisms of ageing and cancer.

## Results

### Transcriptome data metrics

To unveil the potential adaptive mechanisms that allow the long-lived rodent *N. galili* to tolerate severe hypoxia on the gene regulatory level, we subjected *n* = 3 individuals of both species to either 6% O_2_ or to normoxia (room air) for six hours and dissected liver, kidney, and spleen tissues. RNA was isolated to generate transcriptome-wide mRNA expression profiles by Illumina sequencing. Between 10 and 54 million reads per dataset were mapped to their respective reference genomes (Supplementary Dataset S1). For validation, expected organ-specific gene expression patterns as described by Yu et al. (2014) were clearly observable within our data (Supplementary Material Fig. 1). We detected expression in at least one of the biological replicates for 16359 genes in *Spalax* and 17741 genes in rat in all analyzed tissues under both oxygen conditions (Supplementary Dataset S2). When comparing the gene expression for each tissue via principal component analyses (PCA), we observed that replicate samples (*n* = 3) of the same species subjected to the same oxygen conditions clustered together as expected (Supplementary Material Fig. 2). In all tissue comparisons, the interspecies variance between *Spalax* and rat was larger than between the hypoxia- and normoxia-treated samples of the same species, implying that hypoxia induction had less overall impact on the transcriptional profile than species divergence.

### Quantitative transcriptome differences in the hypoxia response of *Spalax* and rat

**Fig. 1:**
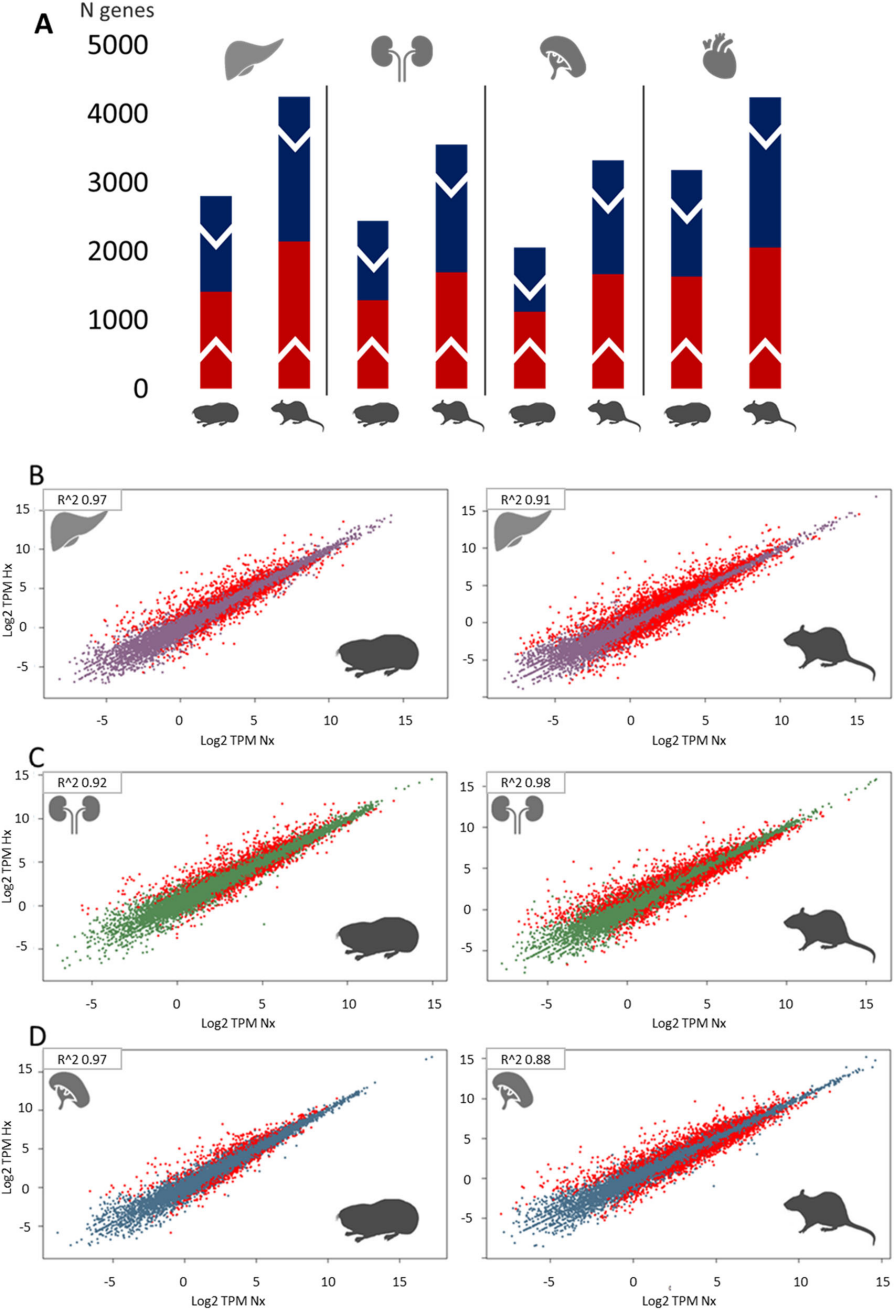
Quantitative transcriptome differences in hypoxia-exposed *Spalax* and rat. **A** Number of *Spalax* and rat DEGs after hypoxic stress exposure in liver, kidney, and spleen. Red = upregulated, blue = downregulated genes. **B**–**D** Scatterplot visualization of DEGs following hypoxic treatment in *Spalax* and rat liver, kidney and spleen. In each graph, log2 TPM fold-changes are plotted. Differentially expressed genes (padj <0.05) are marked in red.

To study the magnitude of the gene regulatory response after hypoxic stress, we identified differentially expressed genes (DEGs) between normoxia and hypoxia for all three tissues in both species (padj. < 0.05). In all tissues, the hypoxia response was notably stronger in rat than in *Spalax* (Fig. 1A). After hypoxia treatment, about 1.5 times more genes were dysregulated in rat liver, 1.45 times more in rat kidney and 1.6 times more in rat spleen, as compared to *Spalax*. Figure 1B–D shows tissue-wise scatterplots of the hypoxia-induced genes for rat and *Spalax*. In particular in spleen, many more dysregulated genes (red dots) were observed in rat (3326) compared to *Spalax* (2055). Together, these data indicate a stronger response to hypoxic stress of rat tissues than in the hypoxia-adapted *Spalax*.

**Fig. 2:**
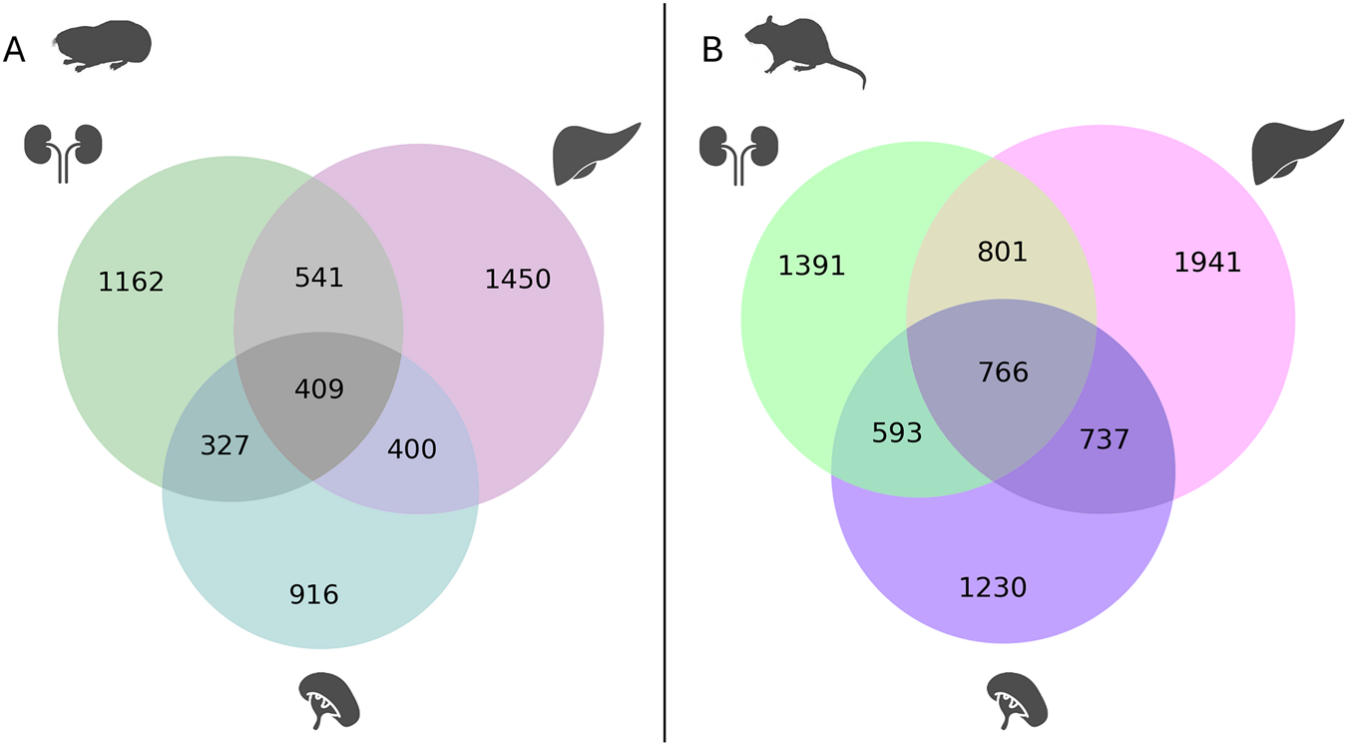
Venn diagrams illustrate the number of hypoxia-regulated genes compared between the three organs studied (padj < 0.05). Genes are counted separately for *Spalax* (**A**) and rat (**B**).

In addition to the observed quantitative differences, the sets of genes induced by hypoxia differed qualitatively between the two species. From those genes differentially expressed under hypoxia in *Spalax*, only 46% (liver), 33% (kidney), and 37% (spleen) were also regulated in the corresponding rat organ (Supplementary Material Fig. 3). This suggests that both organisms transcribe a large set of species-specific hypoxia-responding genes. Ca. 32% (1677) and 39% (2897) of hypoxia-induced DEGs were common to at least two organs in *Spalax* and rat, respectively (Fig. 2). These genes might represent a systemic transcriptional response towards hypoxic stress acting across different tissues.

**Fig. 3:**
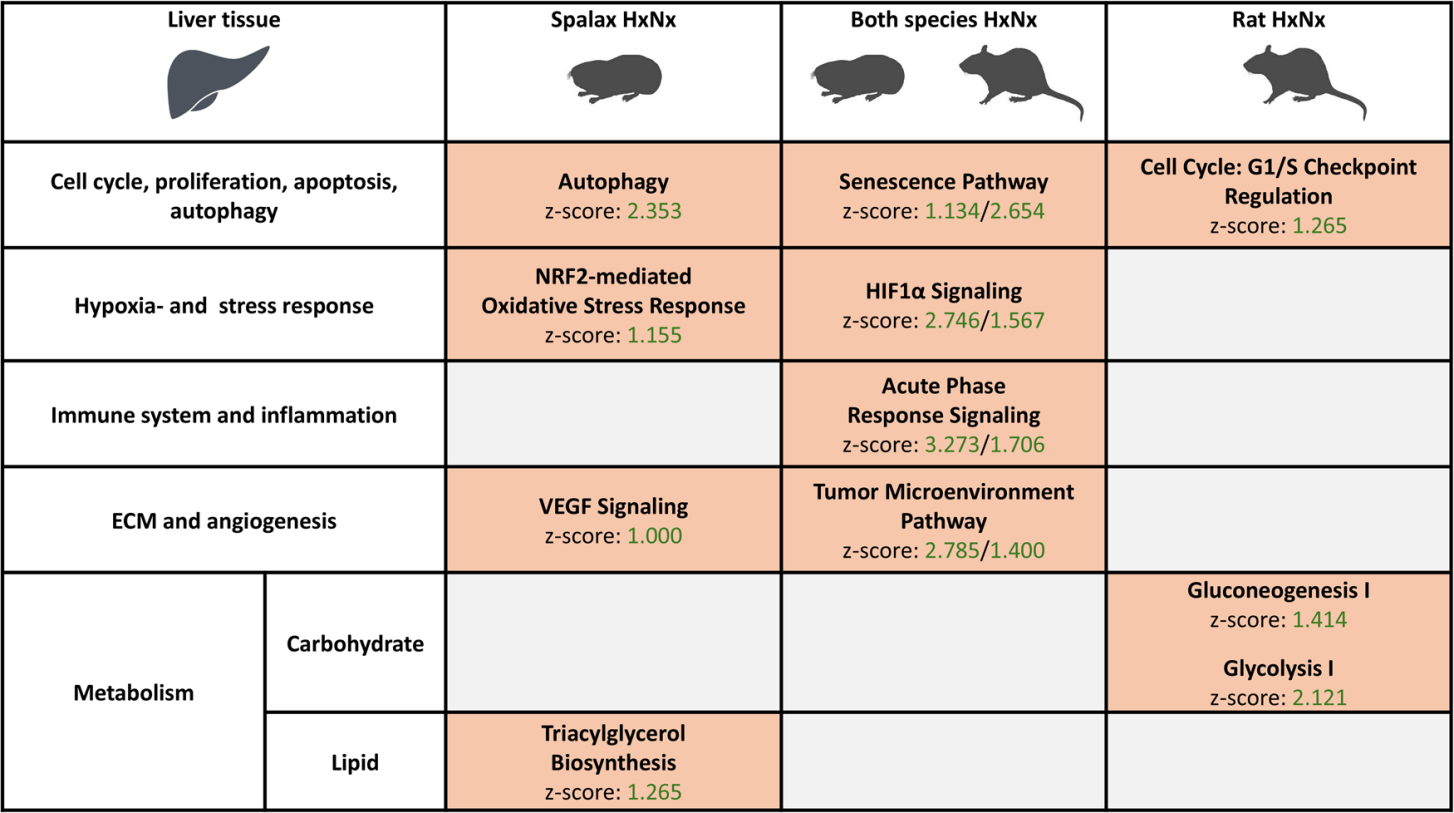
Selected pathways induced by hypoxia in the liver of *Spalax* and rat (padj < 0.05). Pathway enrichment was predicted based on the genes differentially expressed at hypoxia and normoxia (HxNx). Pathways are grouped into larger categories (left margin) for improved overview (orange = predicted activation (z-score > 1).

To interpret the biological function of the hypoxia-responsive genes, pathway enrichment analyses were performed on the DEGs of all three organs in *Spalax* and rat (Supplementary Dataset S3). Identified pathways describing biological processes were summarized for comparison. In **liver** tissue of both species, enriched hypoxia-activated pathways were associated with traits involved in ageing, including cell death (e.g. “Senescence Pathway”), proinflammatory processes (e.g. “Acute Phase Response Signaling”), hypoxia stress response (e.g. “HIF1α Signaling”), extracellular matrix (ECM) and angiogenesis (e.g. “Tumor Microenvironment Pathway”) (Fig. 3). In rat, but not in *Spalax*, enrichment in metabolism-associated terms like “Gluconeogenesis I” and “Glycolysis I” was detected in the hypoxia-activated genes.

**Fig. 4:**
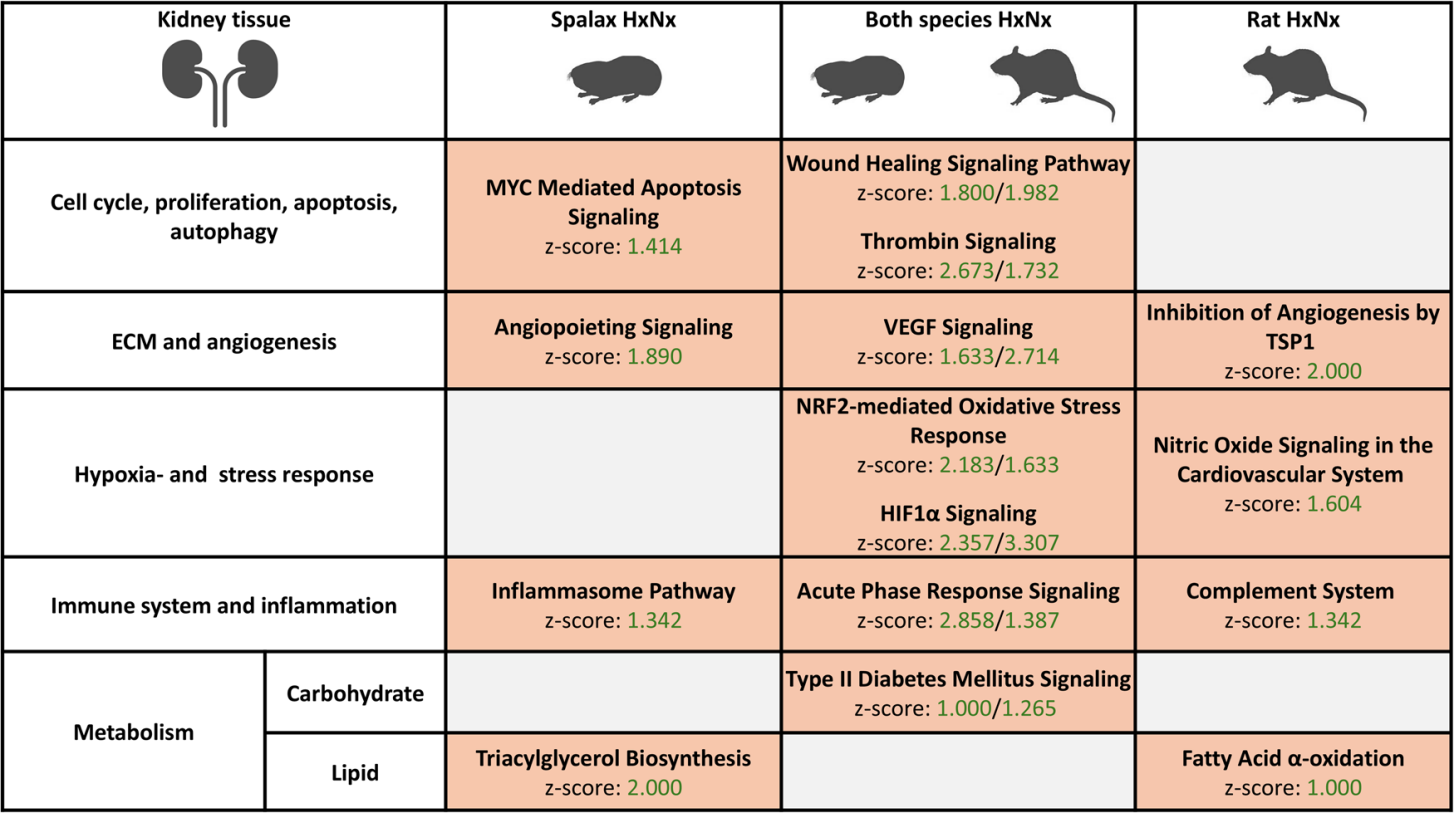
Selected pathways induced by hypoxia in the kidney of *Spalax* and rat (padj < 0.05). Pathway enrichment was predicted based on the genes differentially expressed at hypoxia and normoxia (HxNx). Pathways are grouped into larger categories (left margin) for improved overview (orange = predicted activation (z-score > 1).

In **kidney**, in addition to the regulation of general cellular signaling processes, hypoxic activation of the following pathways was observed in both species (Fig. 4): “Wound healing Signaling Pathway”, angiogenesis and ECM formation (”VEGF-Signaling”, “Tumor Microenvironment Pathway”), hypoxia- and oxidative stress-associated processes (“HIF1α Signaling”, “NRF2-mediated Oxidative Stress Response”), proinflammatory pathways (“Acute Phase Response Signaling”) and metabolism-associated terms (e.g. “Type II Diabetes Mellitus Signaling”). Particularly in *Spalax*, the activation of many ageing- and cell-death-associated terms was detected (e.g., “MYC Mediated Apoptosis Signaling).

**Fig. 5:**
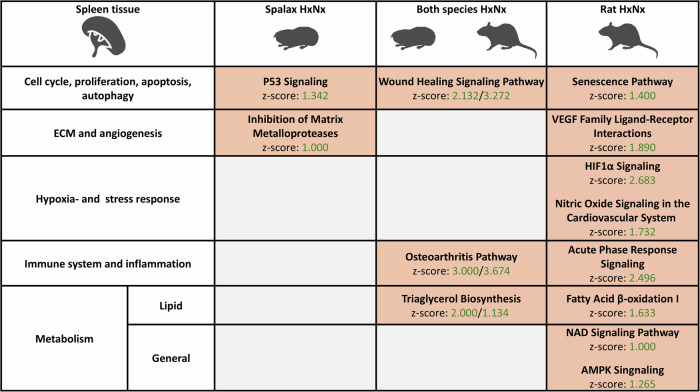
Selected pathways induced by hypoxia in the spleen of *Spalax* and rat (padj < 0.05). Pathway enrichment was predicted based on the genes differentially expressed at hypoxia and normoxia (HxNx). Pathways are grouped into larger categories (left margin) for improved overview (orange = predicted activation (z-score > 1).

In **spleen** tissue of both species (Fig. 5) hypoxia-responsive DEGs were enriched in terms related to the activation of signaling pathways that are associated with pathologies and damage (“Wound Healing Signaling Pathway”, “Osteoarthritis Pathway”, “IL6-Signaling”). In notable contrast to the other organs, hypoxia response pathways (“HIF1α Signaling”,” Nitric Oxide Signaling in the Cardiovascular System”) were specifically activated in rat, but not in *Spalax*.

The results indicated that many processes important for a hypoxia response are activated in both, the adapted rodent *Spalax* and the non-adapted rat. To determine whether this apparent functional similarity in the hypoxia response is orchestrated by the regulation of *the same genes* in *Spalax* and rat, we compared the regulated genes from hypoxia-responsive pathways like “HIF1α Signaling” or “NRF2-mediated Oxidative Stress Response” between *Spalax* and rat in all analyzed organs (Supplementary Dataset S4). In liver and kidney, both species regulated around half of the *n* = 139 analyzed hypoxia-associated genes, while in spleen only *n* = 50 genes were hypoxia-responsive in the rat and an even lower number (*n* = 19) in *Spalax*. Only *n* = 13 genes showed a hypoxia-induced regulation in both species and all three organs (irrespective of their direction of regulation) and might thus fulfill key roles in the stress response (Supplementary Dataset S5). In an organ-wise comparison, *n* = 28 genes in liver, *n* = 21 in kidney and no genes in spleen were regulated in the same direction in both species. On the other hand, *n* = 39 genes in liver, *n* = 40 in kidney and *n* = 42 in spleen were regulated in only one of the two species: e.g., *Hypoxia-inducible factor-2alpha* (Epas1) was only upregulated in rat, but not *Spalax* liver. Among this set were also genes that showed an opposing direction of regulation in *Spalax* and rat like the *MAP kinase-interacting serine/threonine-protein kinase 1* (*Mknk1*), which was hypoxia-upregulated in rat liver, but downregulated in *Spalax*.

In summary, the transcriptome data revealed that around 1.5-fold more genes are regulated in rat compared to *Spalax* in response to hypoxia. The majority of regulated genes differed between *Spalax* and rat, yet this resulted in the activation of similar biological pathways in liver and kidney. In case of the hypoxia and stress-response pathways, many genes showed species-specific response patterns. In spleen, the hypoxia-response was attenuated in both species, but especially in *Spalax*. Our results thus indicate an adapted gene regulatory hypoxia-response in *Spalax*, which involves less (and different) genes.

### Differences in gene expression under *normoxic* conditions between *Spalax* and rat

**Fig. 6:**
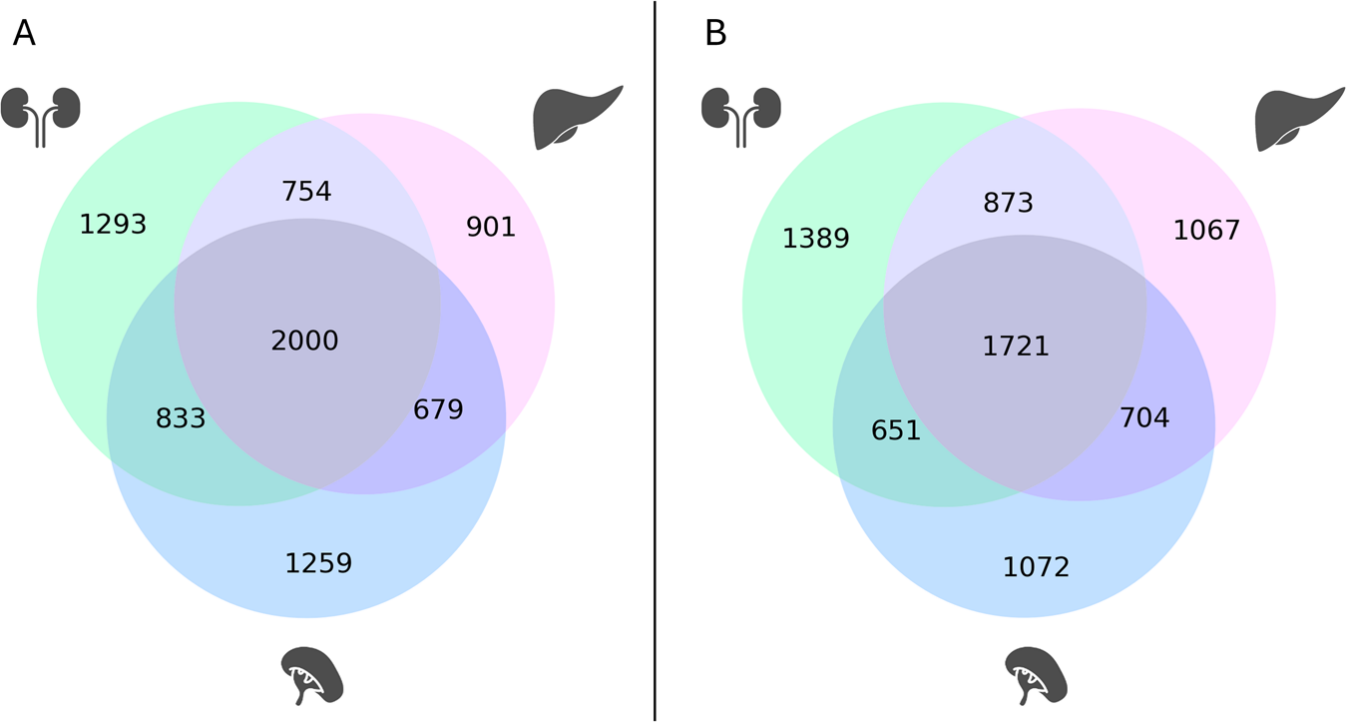
Venn diagram showing the number of differentially expressed genes observed in kidney, liver, and spleen tissue in the interspecies comparison between *Spalax* and rat kept at normoxic conditions. **A** Genes showing higher expression in *Spalax* than in rat. **B** Genes showing lower in *Spalax* than in rat. Criterion for differential expression was a value of padj < 0.05. The large number of genes, which are differentially expressed in all three tissues, indicates across-organ adaptations on the gene regulatory level in *Spalax* compared to rat.

Differences between *Spalax* and rat in gene regulation may not only manifest themselves during an acute response to hypoxic stress, but also “constitutively” at *normoxic* conditions. In all three organs, more than half of the genes were differentially expressed between *Spalax* and rat at normoxia, the number being evenly distributed between higher- and lower-expressed genes (Fig. 6). Similar to the patterns observed in the transcriptional hypoxia response, most genes (54% of all DEGs, *n* = 8215 of 15196) were either up- or downregulated between the two species in at least two organs. Around 26% (*n* = 2000) and 23% (*n* = 1721) of the DEGs were upregulated and downregulated, respectively, in all three *Spalax* vs. rat organs, indicating body-wide adaptations on the gene regulatory level in the two species (Fig. 6).

**Fig. 7:**
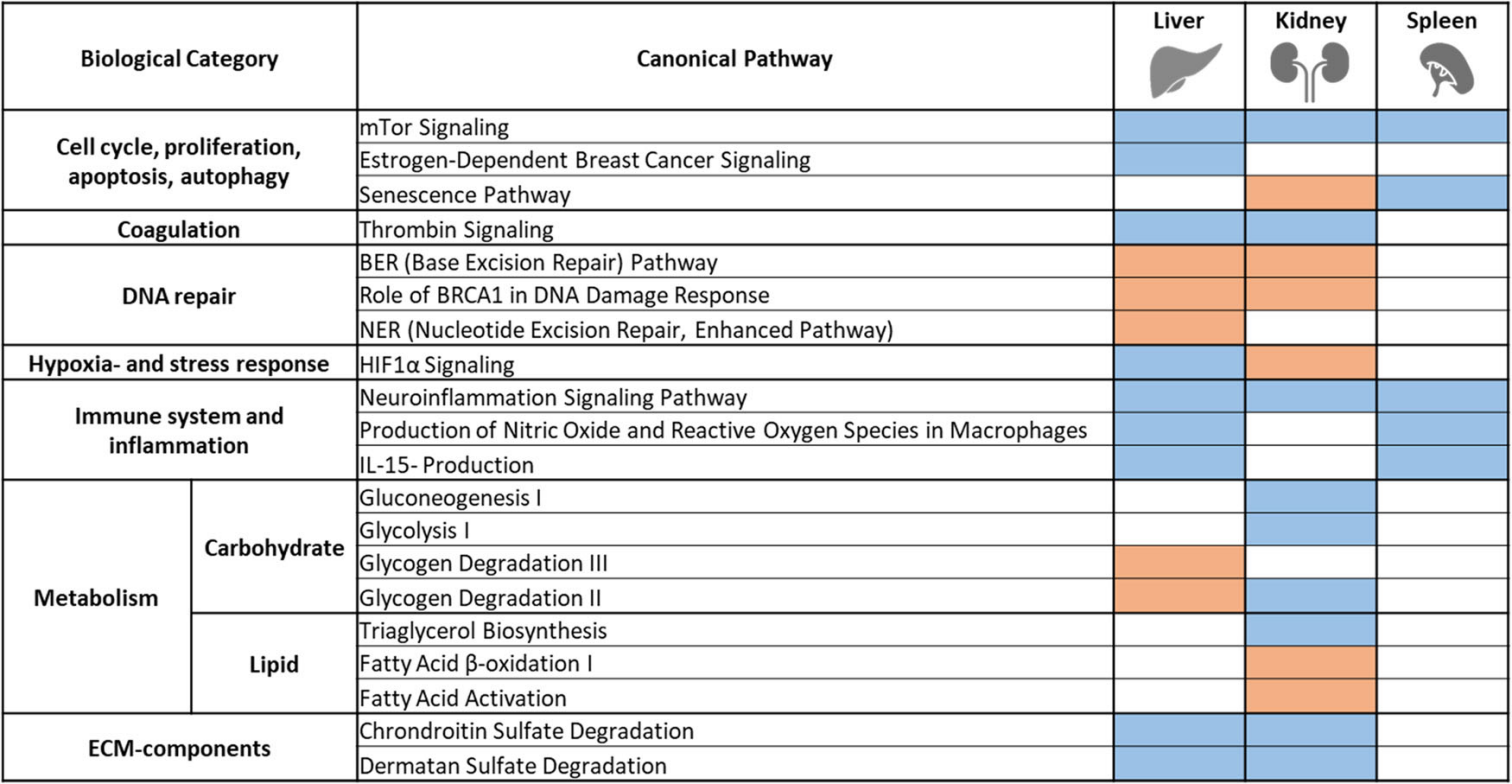
Selected pathways differentially expressed between *Spalax* and rat (padj < 0.05) in normoxic liver, kidney and spleen. Pathways are grouped into larger categories (left margin) for improved overview (orange = predicted activation in *Spalax* (z-score > 1), blue = predicted inactivation in *Spalax* (z-score < −1)).

Interspecies DEGs at normoxia were analyzed for pathway enrichment to interpret their biological function (Supplementary Dataset S3). We observed activation of DNA repair-associated pathways (e.g., “Base excision repair”) in multiple *Spalax* organs, compared to rat (Fig. 7). In parallel, processes associated with proliferation (“mTOR signaling”), proinflammatory pathways (“Neuroinflammation Signaling Pathway”), coagulation (“Thrombin Signaling”) and extracellular matrix components (“Dermatan Sulfate Degradation”) were predicted as inactivated. Enrichment results in *Spalax* **liver** tissue specifically showed activation of metabolic pathways (“Glycogen Degradation II“, “Glycogen Degradation III“), but inactivation of hypoxia-associated pathways (“HIF1α Signaling”). In **kidney**, “HIF1α Signaling” was predicted as activated, while carbohydrate metabolism-associated pathways (“Glycolysis”,” Gluconeogenesis”, “Glycogen Degradation”) were predicted as inactivated. In kidney, we specifically observed enrichment of genes associated with lipid metabolism (“Triacylglycerol Biosynthesis” and “Fatty Acid β-oxidation I”). Notably, in **spleen**, we did not observe activity changes for DNA-repair- or metabolism-associated processes.

**Fig. 8:**
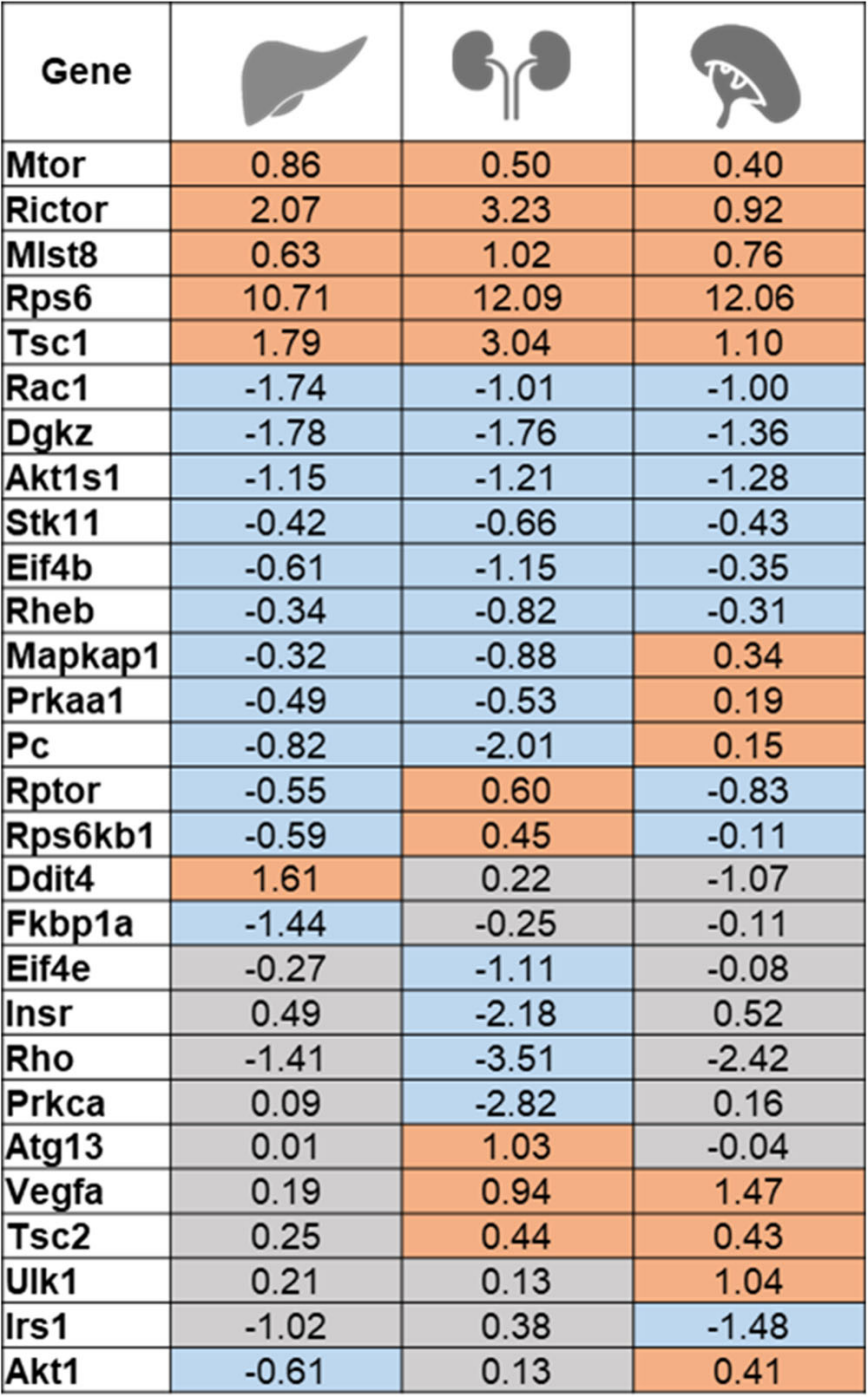
Genes contributing to mTor signaling and their respective fold-changes in differential expression between *Spalax* and rat at normoxia. Orange = elevated expression in *Spalax* compared to rat (Log_2_FC > 0), blue = lower expression in *Spalax* (Log_2_FC < 0), white = differential expression not significant (padj. > 0.05).

In conclusion, the enrichment analysis of pathways differentially expressed between *Spalax* and rat at normoxic conditions pinpointed several processes that showed the same activation status across *Spalax* organs. Detailed inspection of such pathways, as exemplified here by mTOR (Fig. 8), a key metabolic regulator implicated in ageing, in fact revealed that the same genes were differentially expressed in more than one *Spalax* organ with a similar direction and strength of regulation, suggesting that those pathways are regulated body-wide in *Spalax*.

### Validation of the RNA-Seq analysis via qRT-PCR

Based on the results of the functional enrichment analyses and selection of important candidate genes associated with ageing, cancer and hypoxia adaptation, we studied the hypoxia inducibility and interspecies expression differences of a selected gene set representing different functional categories (*A2m, Atr, Cisd2, Fgf21, Wrn, Xpa, Rcan1, Gpnmb, Fen1, Hmox1, Vegfa, Pnkp*) by qRT-PCR (Supplementary Material Tab. 1). We could confirm the direction of all relevant gene expressional changes in the respective organs.

## Discussion

Subterranean rodents like the blind mole rat *(Nanno)Spalax* spec. and the naked mole rat *H. glaber* exhibit a fascinating combination of phenotypes, linking their remarkable longevity and tumor resistance to an adaptation to environmental hypoxia^9,12,13^. Previous studies, addressing single genes and complete genome sequences revealed interesting mutations in candidate coding sequences, but also suggested that differences in gene regulation may underlie adaptations in the subterranean species^13,27,34–36^. Here, we generated and compared RNA-Seq datasets from vital organs of the hypoxia-tolerant blind mole rat *S. galili* and – for comparison – from the hypoxia-sensitive rat. We hypothesized that, in particular, organ-overarching patterns of gene expression might yield insights into the molecular pathways that are responsible for the complex phenotype of *Spalax*. Note as a limitation that, in this study, female *Spalax* individuals were compared to male rats. To identify and remove potential sex biases in gene expression, we analyzed publicly available RNA-Seq datasets from male and female rats (for details see Methods section). Pathways containing more than 10% sex-biased DEGs were then excluded from our subsequent analyses and data interpretation. However, the possibility of residual sex bias in our results cannot be entirely ruled out.

Applying a threshold of 2-fold, the number of differentially expressed genes responding to hypoxia in liver, kidney and spleen of *Spalax* and rat, respectively, ranged between ca. 800 and 2100, in line with previous transcriptome studies of hypoxia-exposed rodents^13,37,38^. At normoxia, 6426 (liver), 7173 (kidney) and 6205 (spleen) genes were significantly differently expressed at least 2-fold (log2fc > 1; padj < 0.05) between the two species (Supplementary Dataset S6). This is mirrored by a liver transcriptome comparison of the hypoxia-adapted naked mole rat and its non-adapted hypoxia-sensitive relative, the guinea pig, reporting differential expression of > 4000 genes^39^. Considering differences in the experimental setup and the closer phylogenetic distance between naked mole rat and guinea pig (39.5 mya) compared to *Spalax* and rat (47.4 mya)^40^, our results on the interspecies expression differences are thus in the expected range. Measurements of RNA levels can serve as a reliable proxy for estimating gene expression and can account for approximately two thirds of the observed protein abundance in mammalian cells^41^. In the naked mole rat, a similar correlation between the RNA and the protein level in an interspecies comparison has been previously reported^39^. However, the same study showed that for some key adaptive processes such as oxidative phosphorylation, differential expression only manifested itself at the protein level. In the future, it will thus be necessary to integrate our transcriptomics approach with protein data to more comprehensively understand adaptation of gene regulation in *Spalax*.

**Fig. 9:**
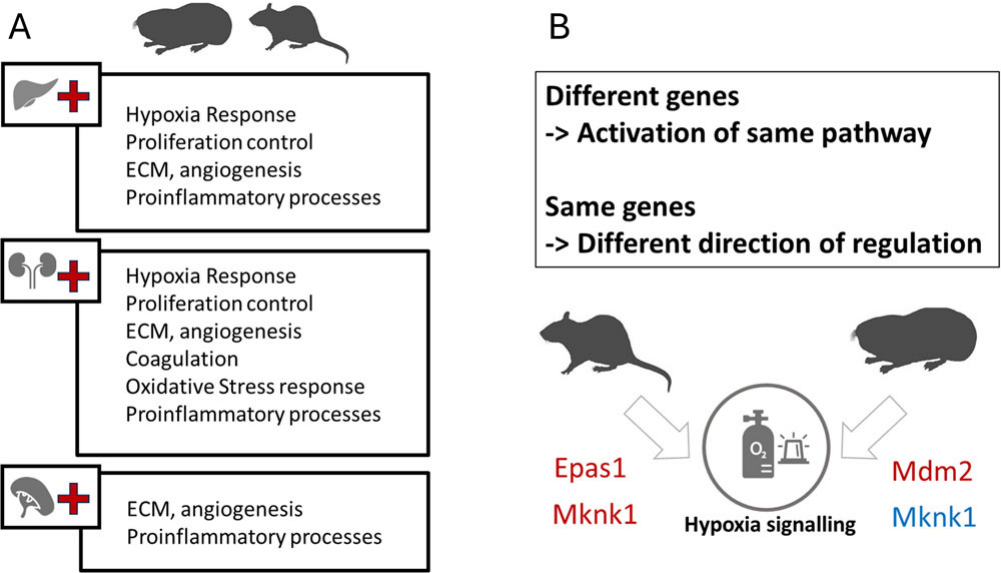
Common and species-specific patterns of transcriptional gene regulation. **A** Often, similar biological pathways are significantly enriched and activated (plus symbol) in the organs of both, Spalax and rat after hypoxic stress. **B** A complex picture emerges when differentially expressed genes within pathways are inspected: expression of *different* genes in Spalax and rat may lead to activation of the *same* pathway, as exemplified by *Epas1* and *Mdm2 (red = upregulation)*. In contrast, the same gene (here: *Mknk1*) can be regulated in opposite directions in response to hypoxia in the two species (blue = downregulation).

As a general trend in all three organs studied, a significantly higher number of genes responded to hypoxic stress in the hypoxia-sensitive rat as compared to the hypoxia-tolerant *Spalax* (comp. Figure 1). At the same time, the majority of hypoxia-responsive genes differed between the two rodents (Supplementary Material Fig. 3). Still, in many cases, pathways associated with similar biological functions such as response to hypoxia, proliferation control, angiogenesis or immune response were activated in both species (Fig. 9). Detailed investigation revealed that often different genes were regulated in the two species within a respective biological pathway, while in other cases the same genes – but showing opposing directions of regulation - were involved in *Spalax* and rat. These results indicate that, while classical hypoxia response pathways are activated in both rodents upon stress exposure, the adapted *Spalax* achieves a biological response by utilizing less genes. The involvement of partially different genes in *Spalax* compared to rat, as exemplified by the mTor signaling pathway, indicates a potentially more sophisticated gene regulatory response in the blind mole rat, which might conserve energy by utilizing less resources for gene expression.

**Fig. 10:**
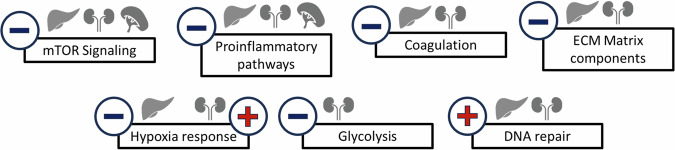
Predicted status of pathways associated with the *Spalax* phenotype at normoxic, unstressed conditions. Activation is designated by a red plus sign, inactivation by a blue minus sign. Note that most pathways are affected in at least two or even three organs, indicating organ-overarching adaptations.

Differential gene expression under *normoxic conditions* is believed to be another important adaptive mechanism in *Spalax*. Elevated expression levels of important master regulators of hypoxic stress^16^ and antioxidant defense^13,32,42^ have been observed in the mole rat, suggesting that such higher expression levels of key genes and pathways, already present at normoxia, might enable *Spalax* to react substantially faster towards an acute onset of severe hypoxic stress, which the rodents meet in their underground habitat during heavy seasonal rainfalls^13,15^ This can clearly be a useful adaptive strategy for biological processes like antioxidative defense or DNA-repair, which guarantee genomic integrity. For other biological processes such as inflammation or energy metabolism, a constitutive activation could be wasteful in energy expenditure and would not be in line with the energy-conserving phenotype of many subterranean species including *Spalax*^14^. It is therefore not surprising that we predicted an inhibition of biological pathways associated with inflammation, energy metabolism or angiogenesis in *Spalax* compared to rat (Figs. 7, 10). These gene-regulatory patterns were in many cases observed in more than one organ, often utilizing the same pathway components (e.g. in mTor signaling; Fig. 8). This indeed confirms the hypothesis that body-wide adaptive gene-regulatory mechanisms are acting in *Spalax* on the transcriptional level.

A more detailed look at the gene regulatory pathways involved in the differences between *Spalax* and rat revealed a number of observations that are of potential biological interest, in particular for the evolution of the complex long-lived phenotype. In both species and all three organs, we observed the enrichment of terms associated with the *control of cell death and proliferation* following hypoxic stress exposure. This is an expected response in sensitive species, since hypoxia often leads to the elimination of potentially damaged cells to prevent carcinogenesis^43–45^. Hypoxia-adapted taxa, in contrast, might want to *avoid* unfavorable cell loss when encountering environmental stress repeatedly. In fact, a tight control of apoptosis has evolved in *Spalax* via mutations in the key tumor suppressor p53, some of which mimic mutations found in P53 of human tumors^28^ or are convergently present in distantly related hypoxia-tolerant species^46^. These changes reduce *Spalax* p53’s ability to induce apoptosis-regulating target genes like *Apaf1* and *Noxa*^28^. In addition, *Mdm2*, a negative regulator of p53, was significantly higher expressed in *Spalax* than in rat in hypoxic muscle, brain, and heart tissue^47^. In our study, we observed a similar overexpression of *Mdm2* in *Spalax* liver and kidney, implying a body-wide involvement of this gene in controlling p53-mediated processes in the blind mole rat. While *Spalax* p53 appears to have a reduced stimulatory effect on pro-apoptotic genes, in vitro reporter gene assays revealed that it over-activates genes associated with cell-cycle arrest like *Cdkn1a* and *Pten*, suggesting an adaptive strategy that promotes temporary cell cycle arrest over apoptosis to prevent excess hypoxia-induced cell loss^28^. Interestingly, we detected a lower transcription of *Pten* and *Cdkn1a* in all three *Spalax* organs, possibly down-modulating p53-induced activation under normoxic conditions. Thus, to improve insight into complex pathways, mutations in master regulators should be interpreted together with mRNA levels of their respective target genes. An additional factor that plays an important role in the cellular response to hypoxia is the calcium influx, which in turn, activates apoptosis and affects the transcription landscape. A study on *Spalax* cultured cells revealed a significantly reduced calcium influx during hypoxia^48^, implying a crucial role of maintaining homeostasis, rather than ‘overreacting’ under stress.

As expected, we observed an activation of classical hypoxia response terms like ***HIF1α signaling*** in liver and kidney in both species. Moreover, this pathway was predicted as already activated under normoxic conditions in *Spalax* kidney. Adaptations in gene regulation in the kidney have previously been proposed to play a role in *Spalax* hypoxia resistance, as the kidney is the major site of erythropoietin production. In this context, an elevated HIF1a and EPO production was measured in *Spalax* kidney^16^, which likely contributes to the mole-rat’s elevated erythrocyte numbers^19^. Under hypoxia, non-adapted rodent species additionally show stress-erythropoiesis in the spleen^38^, and the spleen serves as a reservoir of red blood cells in many diving species^49^. Interestingly, *Spalax* spleen showed a lower transcriptional response to hypoxia and fewer interspecies differences in pathway activation under normoxic conditions, as compared to the other organs. This hints at an obsolescence of splenic stress erythropoiesis in *Spalax* due to its well-adapted erythropoietic physiology.

An important cause of cell and DNA damage and thus cancer- and ageing-related pathologies is **oxidative stress**. While basal levels of oxidative stress can be found in essentially all aerobic organisms, they are substantially increased in subterranean, diving, and high-altitude species^50^. *Spalax* faces acute cyclical changes in oxygen levels in its habitat when performing energy-consuming digging activities in confined tunnels at one moment and entering better-ventilated burrow areas at the next. Moreover, when its underground burrow systems are flooded by heavy rainfall, *Spalax* encounters oxidative stress in cycles of acute severe hypoxia followed by re-oxygenation, which produces harmful ROS^51^. Hypoxia-induced upregulation of anti-oxidant defense genes like *Hmox1* can, therefore be observed in many species in response to hypoxia^52^. Our RNA-Seq data confirm earlier qPCR results^32^ showing elevated mRNA level of *Hmox1* in *Spalax* and rat in response to hypoxia, indicating that despite the many species-specific differences in hypoxia gene regulation, also common, conserved transcriptomic responses exist.

**Fig. 11:**
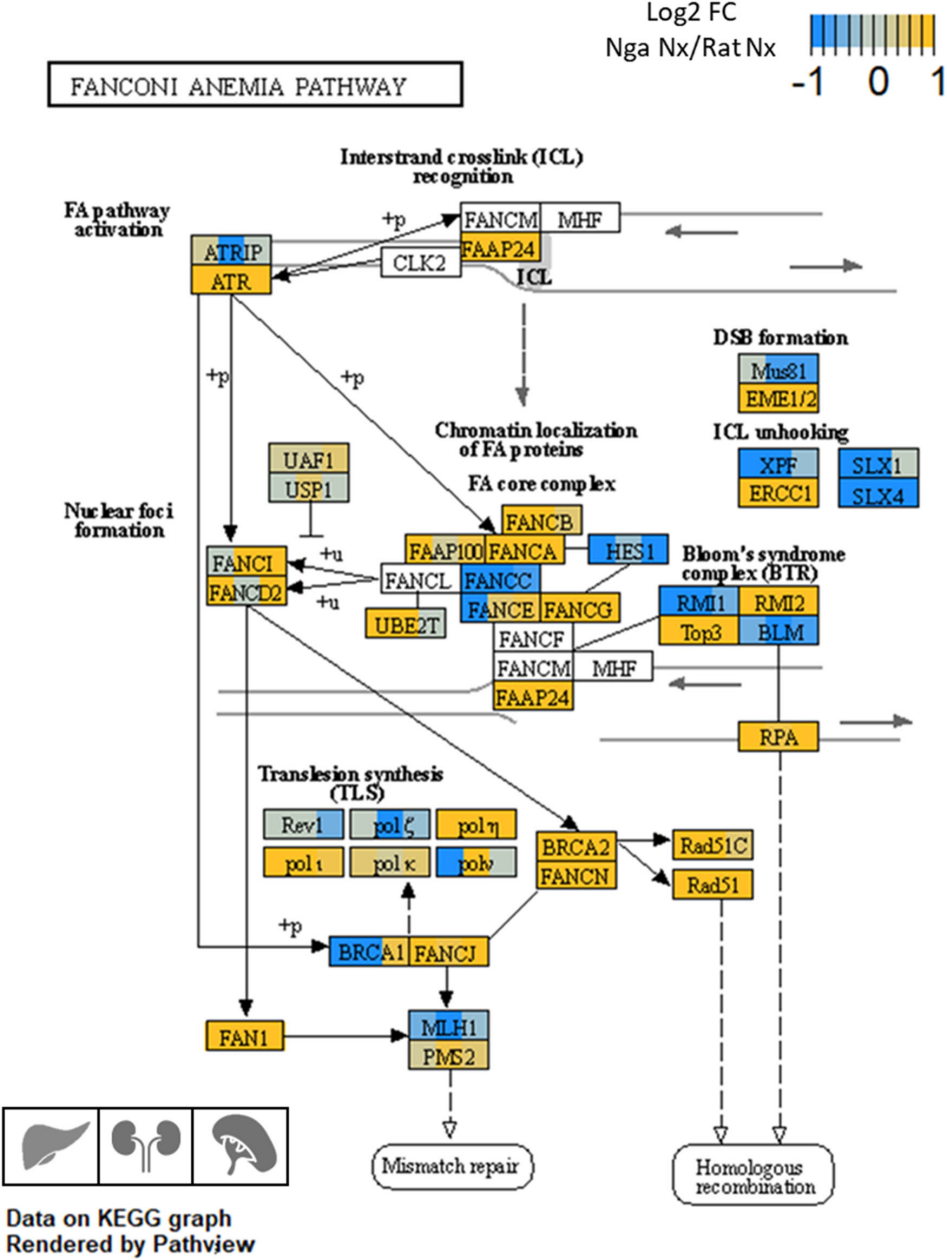
Differential gene expression heatmap for the Fanconi anemia pathway in liver, kidney and spleen of *Spalax* (Nga) and rat (Rno). Respective organs are represented from left to right (liver>kidney>spleen) as squares inside the gene name boxes. Genes with higher expression in *Spalax* than in rat (marked orange) are overrepresented compared to genes with a lower expression in *Spalax* vs. rat. Differential expression is represented as the Log2 FC of expression levels between Spalax and rat at normoxia (Log2 FC (Nga Nx/Rno Nx)).

ROS that are not sufficiently buffered by the radical scavenging system represent a severe threat to genomic integrity, producing various types of DNA damage like nucleotide modifications or crosslinks^53–55^. Furthermore, severe hypoxia induces S-phase arrest and dNTP depletion, leading to additional replicative stress^56–58^. Consistent with this, we observed an enrichment of ***DNA damage response-associated processes*** among the genes that were differentially expressed already at normoxia between *Spalax* and rat. Of note, “Base excision repair” and “Role of BRCA1 in DNA damage response” were predicted as activated in liver and kidney of the mole rat. Previous analyses of RNA-Seq data from *Spalax* liver^13^ and brain^26^ had indicated an elevated expression in the mole rat of key genes from the Fanconi Anemia pathway, which orchestrates different DNA repair activities^59^. In the present study, we could extend these results to kidney and spleen (Fig. 11), which indicates an organ-overarching, body-wide adaptation of DNA repair capacity in *Spalax*. Looking further for candidate genes of special biological relevance, important DNA repair-associated genes like the master activator *Ataxia telangiectasia and Rad3 related (Atr)*, the *breast cancer type 2 susceptibility protein (Brca2)* and the *Werner syndrome ATP-dependent helicase* (*Wrn*) were found overexpressed in *Spalax* liver, brain and spleen compared to rat (present study; as well as in brain tissue^26^, again suggesting body-wide elevated activities of their gene products. Recently, DNA damage repair assays performed on skin fibroblasts isolated from *S. carmeli*, another subspecies of the *S. ehrenbergi* taxon, indeed revealed an elevated resistance against H_2_O_2_, the topoisomerase inhibitor etoposide and UV-C radiation-induced damage compared to rat fibroblasts^60^, suggesting an accelerated repair of those lesions in S*palax*. Since DNA damage is causal to both cancer development^61^) and ageing^62^, we hypothesize that an improved capacity of repairing DNA lesions may substantially contribute to *Spalax’s* anti-cancer and longevity phenotype.

**Fig. 12:**
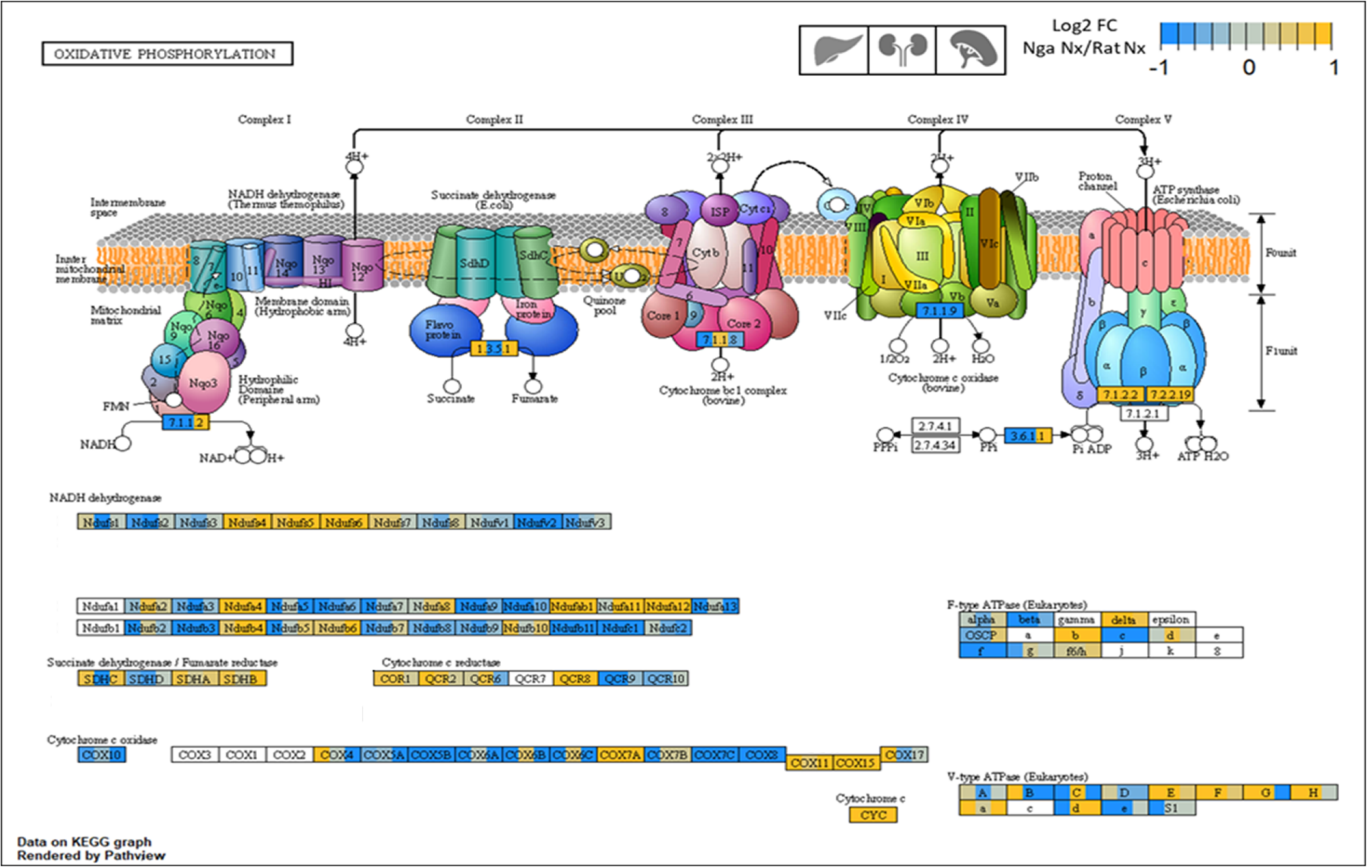
Differential gene expression heatmap of genes contributing to oxidative phosphorylation in liver, kidney and spleen of *Spalax* (Nga) and rat (Rno). Respective organs are represented from left to right (liver>kidney>spleen) inside the gene name boxes. Genes with lower expression in *Spalax* than in rat (blue) are overrepresented compared to genes with a higher expression (orange). Differential expression is represented in form of the Log2 FC of expression levels between Spalax and rat at normoxia (Log2 FC (Nga Nx/Rno Nx)).

An important physiological adaptation to its hypoxic habitat is *Spalax’s* low basal metabolic rate, which reduces its oxygen consumption and, thereby, oxidative stress^17^. Such hypometabolism, which is thought to promote longevity and prevent cancer^63^, was in fact previously inferred from transcriptome analyses of *Spalax* brain, where lower mRNA levels of genes associated with energy metabolism were found compared to rat^13,26^. In support of this, we detected differential expression of genes associated with cellular ***energy metabolism*** in liver, kidney and spleen between *Spalax* and rat at normoxia. For example, key genes contributing to oxidative respiration like *cytochrome c oxidase* showed lower normoxic mRNA expression for most subunits in all analyzed *Spalax* organs (Fig. 12).

Importantly, the transcriptome data predicted an *inactivation* of **mTor signaling** in all three *Spalax* organs compared to rat. Due to its involvement in ageing-related processes like nutrient sensing, proteostasis and mitochondrial dysfunction, the mTOR cascade is considered a major player in life span regulation^64–66^ and mTOR inhibitors like rapamycin are researched for their potential use in lifespan extension^67^. Interestingly, recent studies in the hypoxia-adapted naked mole rat and red-eared slider turtles^68^ suggested an *activation* of mTOR in response to hypoxia, which may aid metabolic reprogramming in favor of anaerobic metabolism. Why this is not observed in *Spalax* remains unclear. However, specific amino-acid replacements in mTORs functional domains were observed in *Spalax* ^13^, suggesting potential adaptations on the functional protein level in the blind mole rat. A constitutive inactivation of the mTOR pathway, as inferred from the transcriptome level in *Spalax*, could mimic the effect of anti-cancer drugs^69,66^ and thus contribute to the mole rat’s cancer-resistance.

Hypoxia also plays a key role in the regulation of **immunity and inflammation** since it is a hallmark of inflamed, infected or damaged tissue^70^. In this context, inflammation can induce the activity of hypoxia-response pathways, and hypoxia may modulate inflammatory signaling^71^. It is therefore not surprising that we detected an enrichment of genes associated with the regulation of inflammatory processes among the hypoxia-responsive genes in all organs of both species. The same was observed in brain tissue in a previous study^72^. Sterile inflammation, which is induced by the secretome of senescent cells, consisting of inflammatory cytokines, chemokines and growth factors, knowingly plays a role in age-related disorders and cancer^73,74^. Stress assays in *Spalax* fibroblasts suggest that the positive feedback loop of IL1α-NF‐κB, which is an upstream regulatory machinery of the inflammatory response, appears to be impaired^75^. Accordingly, we detected in all analyzed *Spalax* organs a decreased expression of proinflammatory factors, some of which are also part of the secretome of senescent cells (e.g. Il1a, Il6r and Cxcl1). We conclude that a lower expression of proinflammatory genes under normoxic conditions in healthy organs may produce an “inflammation-free” phenotype in *Spalax*, possibly contributing to the species’ longevity.

As an important *caveat* to our (and other) transcriptomic interspecies comparisons, it might be argued that the observed differences between taxa simply reflect phylogenetic distance, but not necessarily adaptive processes. A way out of this analytical dilemma can be the inclusion of additional species with and without the studied phenotypes, which may reveal instances of phenotypic convergence, and thus indirectly point at “adaptation in action”. In rare cases, convergent adaptation is realized by ‘strict convergency’, e.g., by mutations affecting *the same* gene in two species adapted to the same selective pressure. One example is the presence of functionally similar amino acid replacements in the proton-gated nociceptor sodium channel Nav1.7 (Scn9a) of *Spalax* and several other species, which are adapted to hypercapnic environments^5^. More often, convergency is realized on a functional level by sometimes utilizing different genes (but from the same pathways) to achieve similar phenotypes. In the context of hypoxia, adapted species that also possess a longevity phenotype like naked mole rats or whales^76^ consistently show differential expression in genes associated with processes like oxidative stress response and ROS defense, metabolism, and DNA-repair compared to non-adapted, related taxa,^34,39,77,78^. Since we observed gene-regulatory changes influencing the very same biological processes in *Spalax*, we infer that these differences in gene expression are, to a high degree, indeed adaptive. There are even examples for strict convergence in overexpression of the same genes in *Spalax* and naked mole rat, as exemplified by the antiprotease alpha2-macroglobulin A2M^13,79^ and DNA-repair genes like ATR^34^. Studying and comparing functional and direct convergencies in adapted species like *Spalax* and naked-mole rat can thus provide valuable insight into the evolution of complex phenotypes and help to pinpoint different ways for modulating physiological processes like hypoxia response, tumor inhibition and geroprotection.

We conclude that the complex cancer- and ageing-resistant phenotype of *Spalax* is likely influenced by transcriptomic changes, which act overarchingly across vital organs and tissues. These transcriptomic changes involve genes from fundamental biological processes like the control of cell death, ROS defense, DNA repair, energy metabolism, immune response, and angiogenesis. Many of these processes link the transcriptional hypoxia response to pathways determining the defense against cancer, the maintenance of genomic integrity, and the emergence of longevity. Our data thus provide additional evidence for the hypothesis that these biomedically most interesting phenotypes have evolved as consequences of *Spalax’*s adaptation to environmental hypoxia. Future studies on cis- and trans- regulatory mechanisms orchestrating adaptation on the gene regulatory level in *Spalax* will provide further insights in the evolution of the blind mole rat’s remarkably complex phenotype.

## Materials and Methods

### Sample preparation

Six *N. galili* specimens were captured from basalt, heavy soil in fields of northern Israel and kept in the animal house of the *Institute of Evolution, Haifa University* in accordance with the regulations of the Israel *Nature and Park Authority, Science and Conservation Unit*. To investigate the impact of O_2_ deprivation on the transcriptomes, three *Spalax* (approximately 4 years old females, 150–250 g) and three rat individuals (Sprague Dawley, 4 month old males, 350–400 g) were exposed to 6% O_2_ for six hours in 70x70x50 cm chambers divided into separate cells at a gas flow rate of 3.5 l/min. No animals died during hypoxia exposure. Afterwards, they were sacrificed immediately by Ketaset CIII (Fort Dodge, USA) injection. From each individual, the kidney and spleen were dissected. Liver samples from the same animals were analyzed prior to this study^13^. Animal experiments were approved by the University of Haifa Ethics Committee (Permit #193/10). RNA was extracted from the kidney and spleen with the RNeasy lipid tissue kit (Qiagen, Hilden, Germany), including an RNase-Free DNase I treatment. RNA integrity was validated on a 2100 Bioanalyzer with the Agilent RNA 6000 Nano kit (Agilent Technologies, Santa Clara, USA). Sequencing libraries were generated with the Illumina TruSeq RNA library Prep kit v2 (StarSEQ, Mainz, Germany) and sequenced as 100 bp paired-end reads on an Illumina HiSeq2500 (Biology Department, Johannes Gutenberg University Mainz, Germany). Sequence data were uploaded for public availability to ENA (Acc. No. PRJEB64658).

### RNA-Seq analyses

RNA-Seq data were quality-trimmed and filtered for sequencing adapter sequences with Trimmomatic v.0.3.6^80^ using a sliding window approach with a window size of 4 nt and a quality score cutoff of 20. The first 13 nt of each read were cropped, and reads shorter than 20 bp discarded. The processed reads were mapped with the STAR aligner v.2.5.3^81^ to the annotated reference genomes of *Rattus norvegicus* (6.0.92; https://ftp.ensembl.org/pub/release92/fasta/rattus_norvegicus/dna/Rattus_norvegicus.Rnor_6.0.dna.toplevel.fa.gz) and *N. galili* (1.0.92; https://ftp.ensembl.org/pub/release92/fasta/nannospalax_galili/dna/Nannospalax_galili.S.galili_v1.0.dna.toplevel.fa.gz). Count data files and index files were generated using an sjdbOverhang of 87. The mismatch maximum was set to 0.04 per base, and a multimap filter was applied with a maximum of 20 matches. Gene IDs of *Spalax* and rat were matched by identifying orthologue Ensemble IDs with *BLASTP* (BLOSUM45 matrix; cutoff length 100 amino acids; minimum identity 30%). Only genes that matched the top BLAST hits as judged by the E-value for both search directions, i.e., *Spalax* query searches in rat databases and vice versa, were used.

Differential gene expression analysis between samples was conducted with DeSeq2 v.1.30.1^82^. Statistical significance was determined by applying the default Wald-test of DESeq2. A padj. cutoff of < 0.05 was used to define differential gene expression. For interspecies comparison, counts were normalized with regard to the exon sum length using the *normMatrix* function as part of the DESeq2 package. Differentially expressed genes were mapped onto relevant *KEGG pathways* for visualization using the *pathview* R package^83^. Principal component analyses were generated with R, using the function plotPCA. Resulting gene lists were compared between species and between different O_2_-conditions using the R VennDiagram package^84^. We validated organ specificity by projecting our gene lists on known, organ-specific gene expression patterns^85^. RNA-Seq data were analyzed with the use of QIAGEN Ingenuity Pathway Analysis (QIAGEN Inc., https://digitalinsights.qiagen.com/IPA^86^); A padj. cutoff of *p* < 0.05 was used to define pathway enrichment and a z-score cutoff of < −1 or > 1 for inactivation and activation, respectively.

A previously published liver RNA-Seq dataset ^13^ was re-evaluated using the same analytical tools and parameters to adequately compare the liver transcriptome to kidney and spleen. Potential biases in liver gene expression (sex, species, strain, age) were already accounted for by Schmidt et al. (2017) via comparison with public rat, mouse, and human RNA-Seq data. Based on these comparisons, a substantial effect of such biases on interspecies- and hypoxia-related gene expression changes was considered improbable. In the present work, we repeated and extended this analysis, in particular to identify the potential influence of sex-biased gene expression. Public RNA-Seq data of kidney, liver, and spleen tissue of n = 4 male and *n* = 4 female rats^85^ were processed in the same way as in our interspecies and hypoxia-normoxia comparisons in order to identify sex-biased genes. A gene was deemed sex-biased if it met the following criteria: padj < 0.05; |Log2FC|> 1. We then inspected the enriched pathways, which emerged in our interspecies and hypoxia-related comparisons (see Results), for the presence of differentially expressed genes (DEGs) with sex-bias. In the liver, 8 out of the 122 significantly enriched pathways contained more than 10% (and up to 17%) sex-biased DEGs. In kidney and spleen, only 5 out of 111 and 0 of 70 significantly enriched pathways met this criterion of sex-biased expression. Conversely, between 93% and 100% of the significantly enriched pathways in each tissue contained less than 10% of sex-biased DEGs (Supplementary Dataset S7). The few pathways that contained more than 10% sex-biased DEGs were not further discussed, and we therefore consider that our main conclusions are not confounded by marked sex-biased gene expression.

### Validation of RNA-Seq data by qRT-PCR

The differential expression of a subset of candidate genes was validated by quantitative real-time reverse-transcriptase PCR (qRT-PCR). 600 ng of quality-checked RNA of all samples was used to synthesize cDNA with the Superscript III RT kit (Thermo Fisher, Waltham, USA). To generate standard-curves for absolute RNA copy number quantification, plasmids containing corresponding amplicons were generated: PCRs were conducted in a peqSTAR 96 Thermocycler (Peqlab, Erlangen, Germany) with the TrueStart Taq DNA Polymerase kit (Thermo Fisher, Waltham, USA) and purified with the Wizard SV Gel and PCR clean up System (Promega, Madison, USA). PCR products were cloned into pGem-T Easy vectors (Promega), transformed in *E. coli* DH10B cells, plated out and purified with the *GeneJET Plasmid Miniprep Kit* (Thermo Fisher). Plasmid inserts were verified by Sanger sequencing (StarSEQ, Mainz, Germany). qRT-PCR was conducted with the GoTaq qPCR Master Mix (Promega) at a total volume of 10 µl in an ABI 7500 Fast Real Time PCR cycler (Applied Biosystems). Serial 10-fold dilutions of plasmids were applied for absolute quantification of samples, measured in triplicates.

## Supplementary information

Supplementary material

Data Set 1

Data Set 2

Data Set 3

Data Set 4

Data Set 5

Data Set 6

Data Set 7

## Data Availability

Sequencing data have been deposited in the European Nucleotide Archive (ENA) under project accession number PRJEB64658.
