# Author Correction: Adaptation of the *Spalax galili* transcriptome to hypoxia may underlie the complex phenotype featuring longevity and cancer resistance

**DOI:** 10.1038/s41514-025-00225-0

**Published:** 2025-05-03

**Authors:** Gesa Poetzsch, Luca Jelacic, Leon Dammer, Sören Lukas Hellmann, Michelle Balling, Miguel Andrade-Navarro, Aaron Avivi, Imad Shams, Anne Bicker, Thomas Hankeln

**Affiliations:** 1 Molecular Genetics & Genome Analysis, Institute of Organismic and Molecular Evolution, Faculty of Biology, Johannes Gutenberg-University, Mainz, Germany; 2 Computational Biology and Data Mining Group, Institute of Organismic and Molecular Evolution, Johannes Gutenberg University, Mainz, Germany; 3 Institute of Evolution, University of Haifa, Mount Carmel, Haifa Israel; 4 Department of Evolutionary and Environmental Biology, Faculty of Natural Sciences, University of Haifa, Mount Carmel, Haifa Israel; 5 Present Address: Nucleic Acids Core Facility, Faculty of Biology, Johannes Gutenberg-University, Mainz, Germany; 6 Present Address: Department of Medicine I, Johannes Gutenberg University Medical Center, Mainz, Germany

**Keywords:** Molecular biology, Transcription

Correction to: *npj Aging* 10.1038/s41514-025-00206-3, published online 6 March 2025

In this article, the panel for Fig. 2B was incorrect and did not accurately reflect the results described in the text. The original article has been corrected.

