# Supplementary material for: Adaptation of the Spalax galili transcriptome to hypoxia may underlie the complex phenotype featuring longevity and cancer resistance
Source: NPJ Aging. 2025 Mar 6;11(1):16. doi: 10.1038/s41514-025-00206-3 (PMC11882797; doi:10.1038/s41514-025-00206-3)
Supplement: Supplementary file 1 — Supplementary material [file 41514_2025_206_MOESM1_ESM.pdf]

Supplementary Figure 1

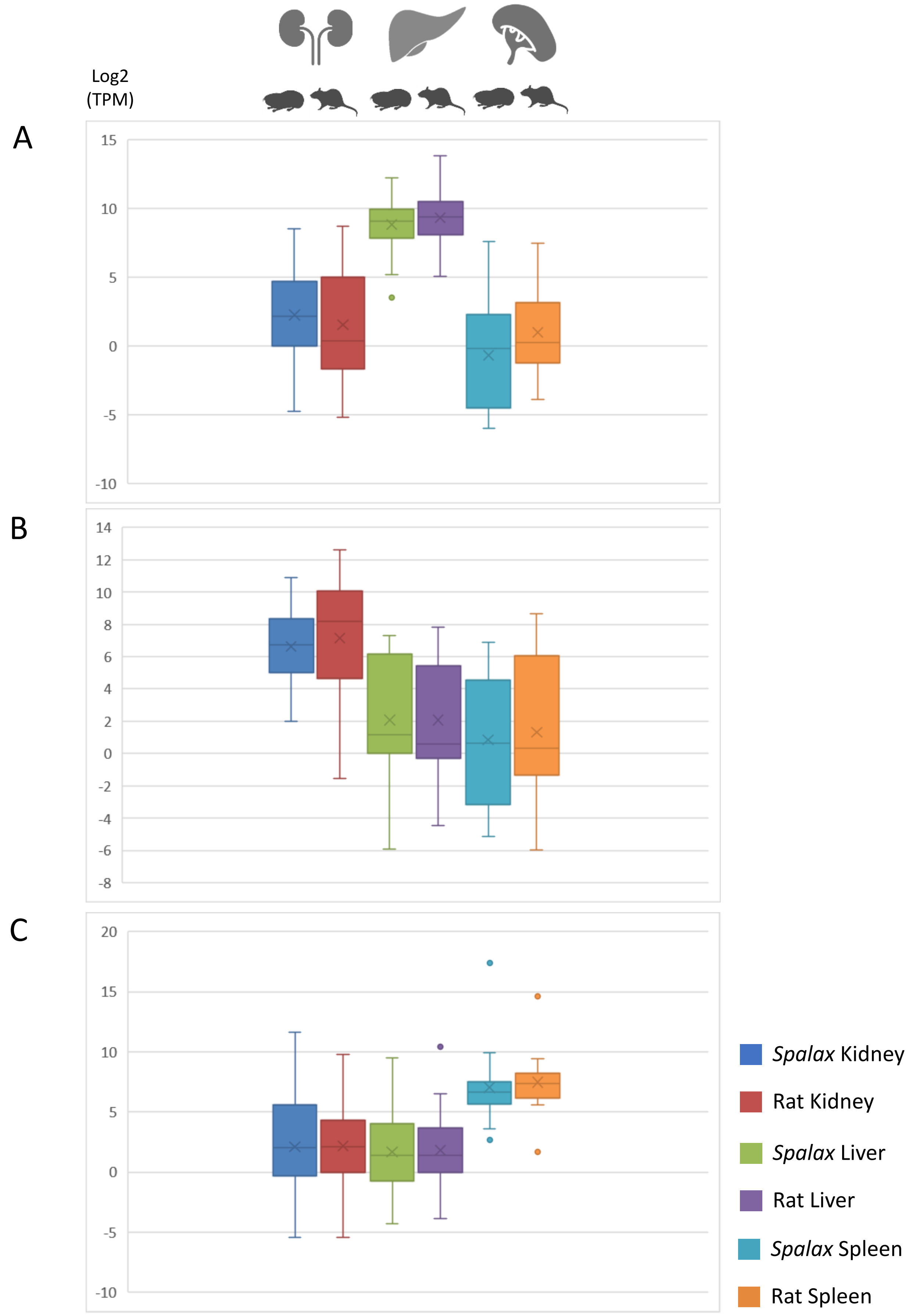

Expression of organ specific genes (Yu et al., 2014) in *Spalax* and rat. A = Liver-specifically expressed genes, B = Kidney-specifically expressed genes, C = Spleen-specifically expressed genes

Supplementary Figure 2

A

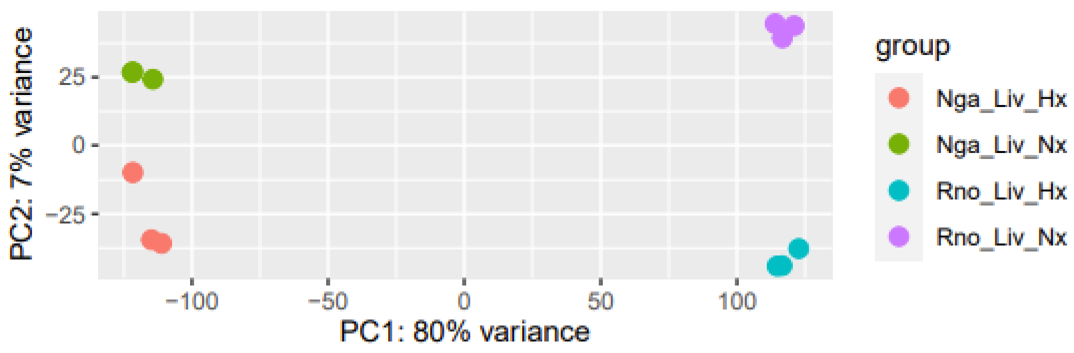

B

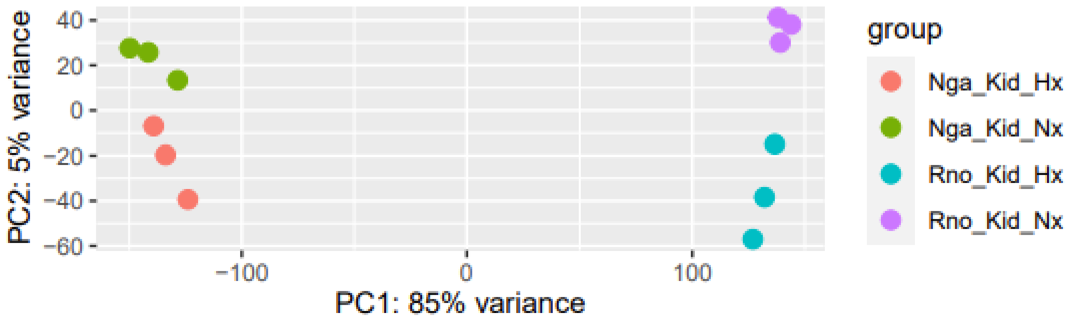

C

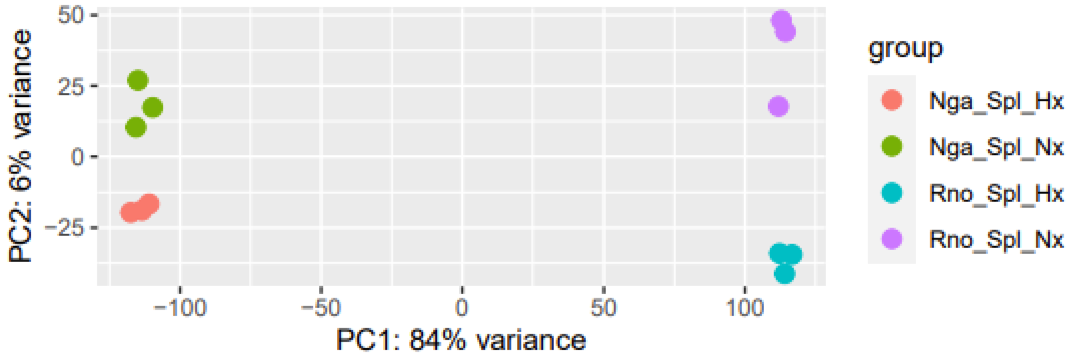

Principal component analyses of trancriptome data of *Spalax* (Nga) and Rat (Rno) under normoxic- (Nx) and hypoxic (Hx) conditions in A = Liver (liv), B = Kidney (Kid) and C = Spleen (Spl)

Supplementary Figure 3

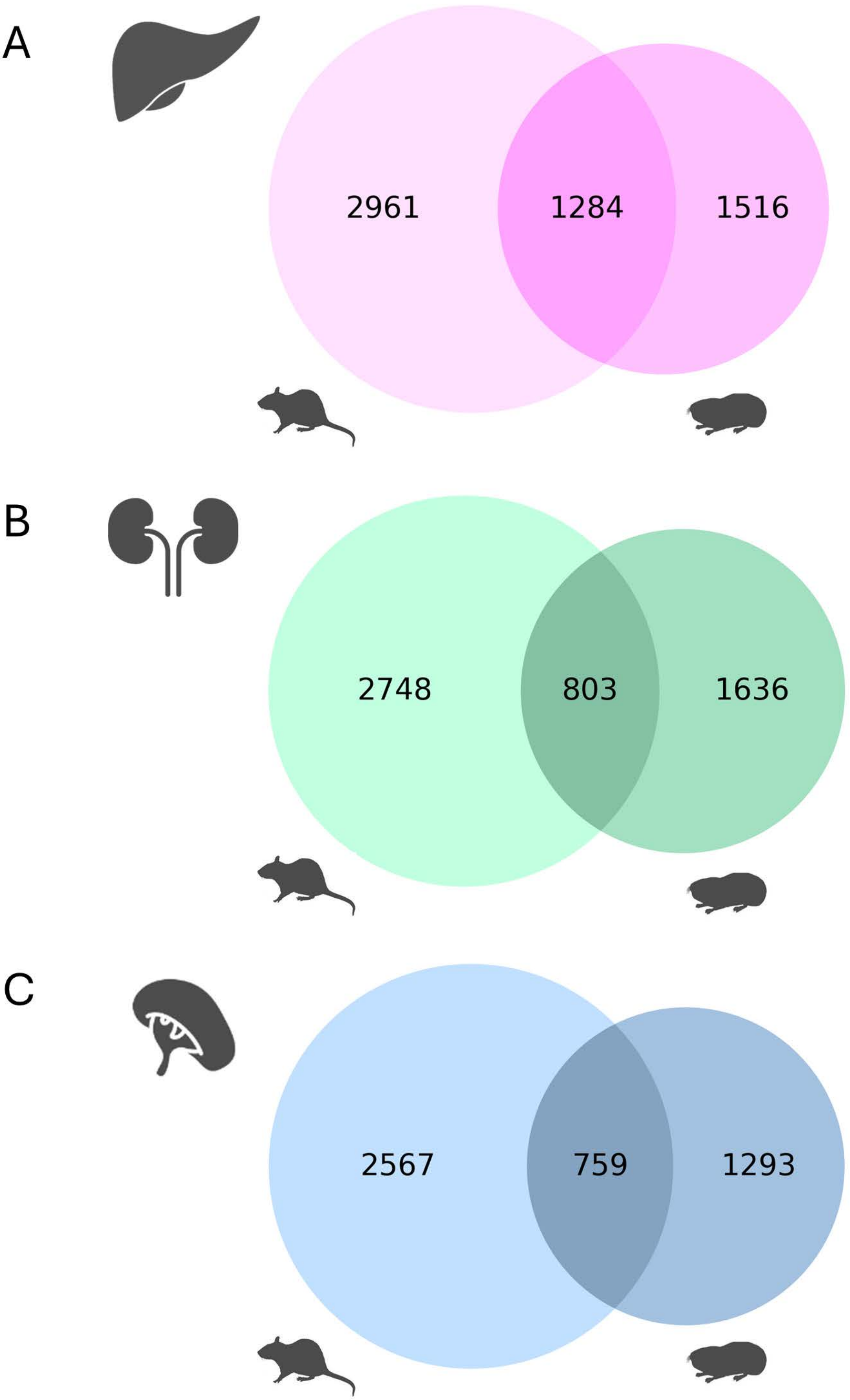

Hypoxia regulation of gene expression compared through all organs (padj < 0.05). Genes differentially expressed under hypoxia in Spalax and rat in A liver, B kidney, C Spleen

Supplementary Table 1

Relative expression of selected candidate genes between hypoxic (Hx) and normoxic (Nx) *Spalax galili* and *Rattus norvegicus* kidney, spleen and liver samples quantified by qRT-PCR and RNA-Seq. Green shading indicates matching results, grey shading indicates diverging results, *= padj. < 0.05, # = padj. > 0.05 low = high ratio due to low copy number at normoxia,

**Kidney**	**Spalax Hx/Nx**		**Rat Hx/Nx**		**Spalax Nx/ Rat Nx**	
**Gene**	qRT-PCR	RNA-Seq	qRT-PCR	RNA-Seq	qRT-PCR	RNA-Seq
Gpnmb	1.13	2.04*	0.82	2.12*	2.63	3.92*
Fen1	1.36	0.34	0.53	0.17	1.78	3.1*
Wrn	-0.59	-0.42	0.85	0.34	3.94	1.73*
Hmox1	4.01	4.7*	3.17	4.12*	7.28	3.15*

**Spleen**	**Spalax Hx/Nx**		**Rat Hx/Nx**		**Spalax Nx/ Rat Nx**	
**Gene**	qRT-PCR	RNA-Seq	qRT-PCR	RNA-Seq	qRT-PCR	RNA-Seq
Vegfa	0.04	-0.11 #	2.68	2.46*	2.16	1.47*
Fen1	-3.54	-0.01	-0.11	-0.02	3.04	1.6*
Wrn	-2.3	-0.45	-0.45	-0.43	4.13	2.44*
Pnkp	-0.86	-0.16	-0.63	-0.64*	2.72	0.71*

**Liver**	**Spalax Hx/Nx**		**Rat Hx/Nx**		**Spalax Nx/ Rat Nx**	
**Gene**	qRT-PCR	RNA-Seq	qRT-PCR	RNA-Seq	qRT-PCR	RNA-Seq
A2m	0.49	0.74	6.56	3.9*	10.57	15.37*
Atr	0.26	-0.06 #	0.58	0.65*	4.84	2.89*
Cisd2	0.38	-0.14 #	0.77	0.56	5.28	2.05*
Fgf21	8.18	7.13*	0.77	-0.54 #	0.14	-5.47*low
Wrn	0.26	0.02	-1.00	-0.37	5.30	2.39*
Xpa	-0.15	-0.45	-0.74	-0.70*	4.63	3.33*
Rcan1	3.41	2.12*	-1.32	-1.70*	-0.22	-1.24*

Supplementary Figure 4

A

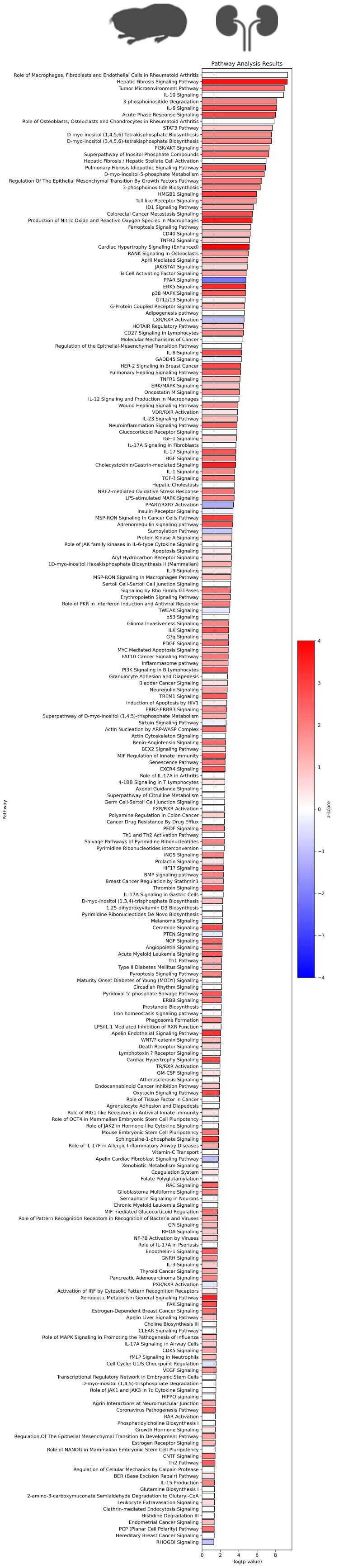

B

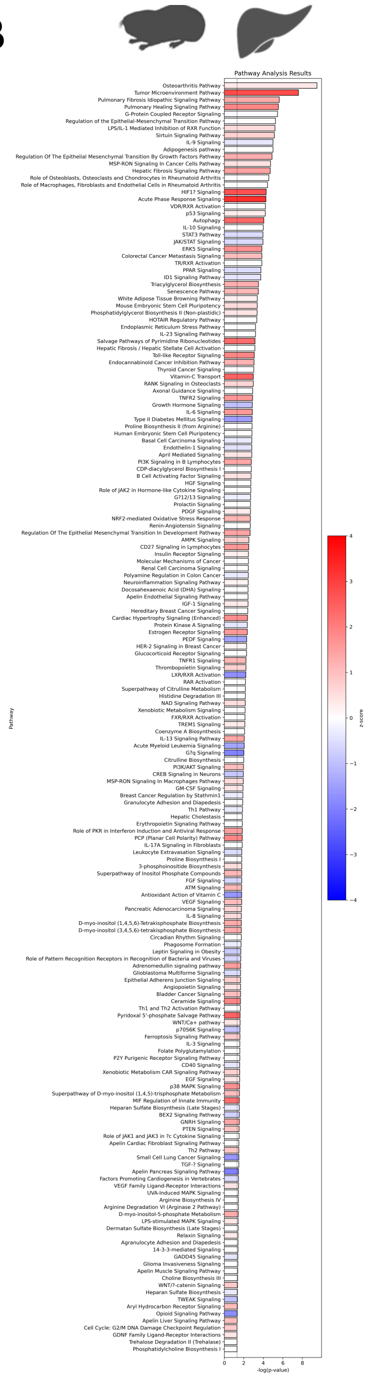

C

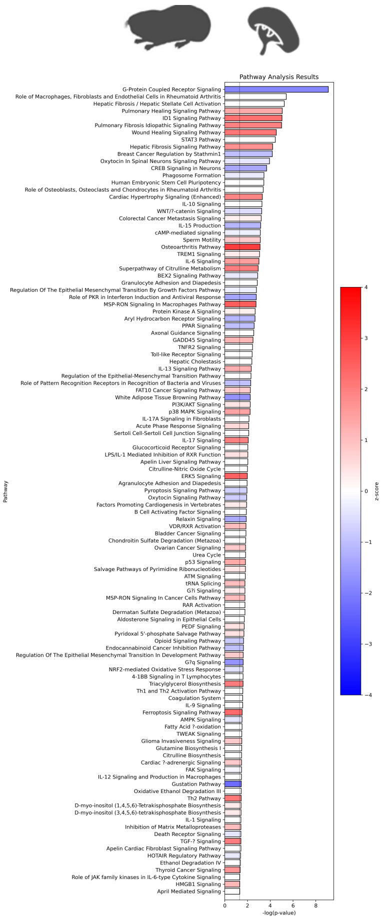

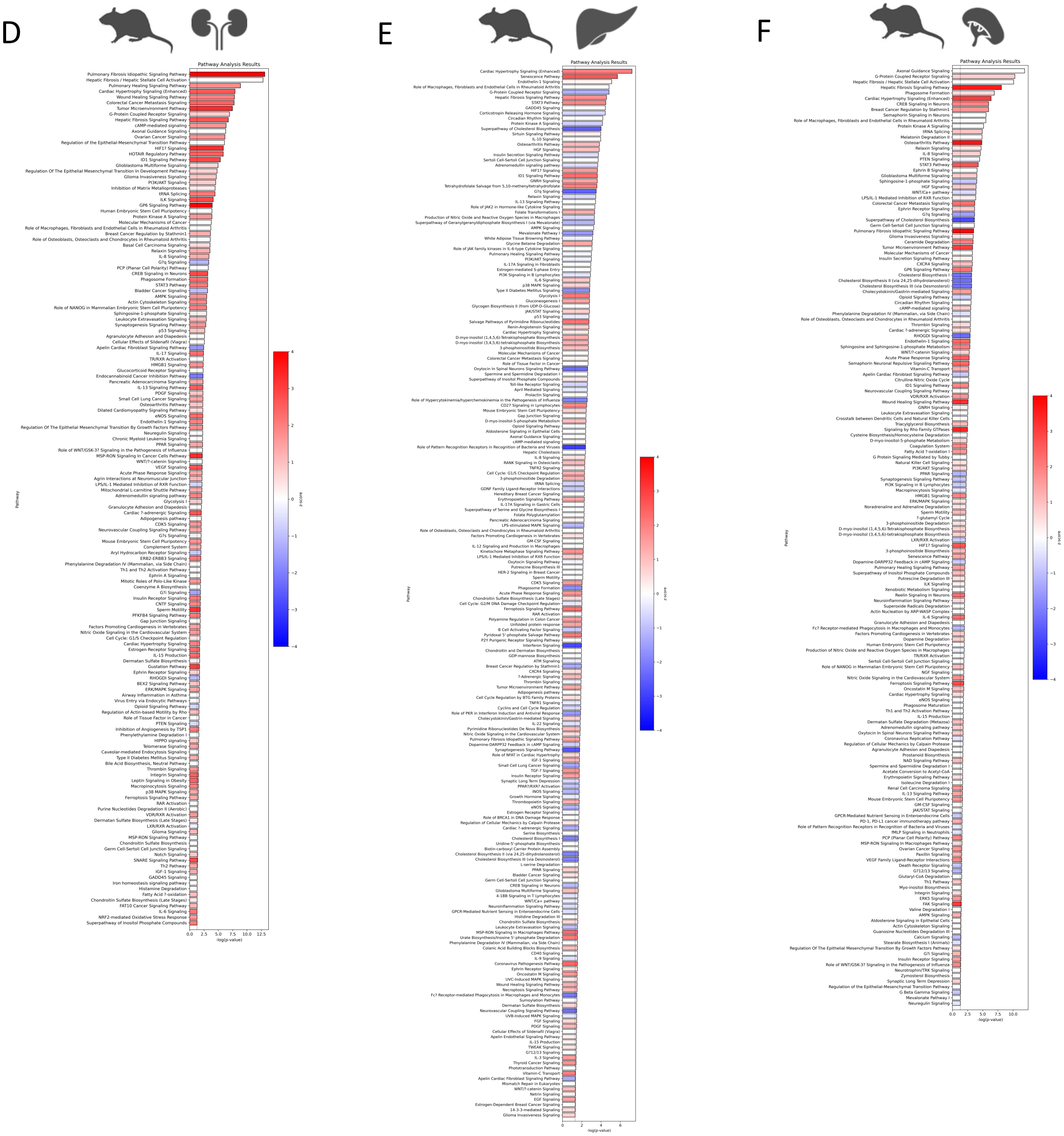
D
E
F
Pathway Analysis Results
Pathway
-log(p-value)
z-score

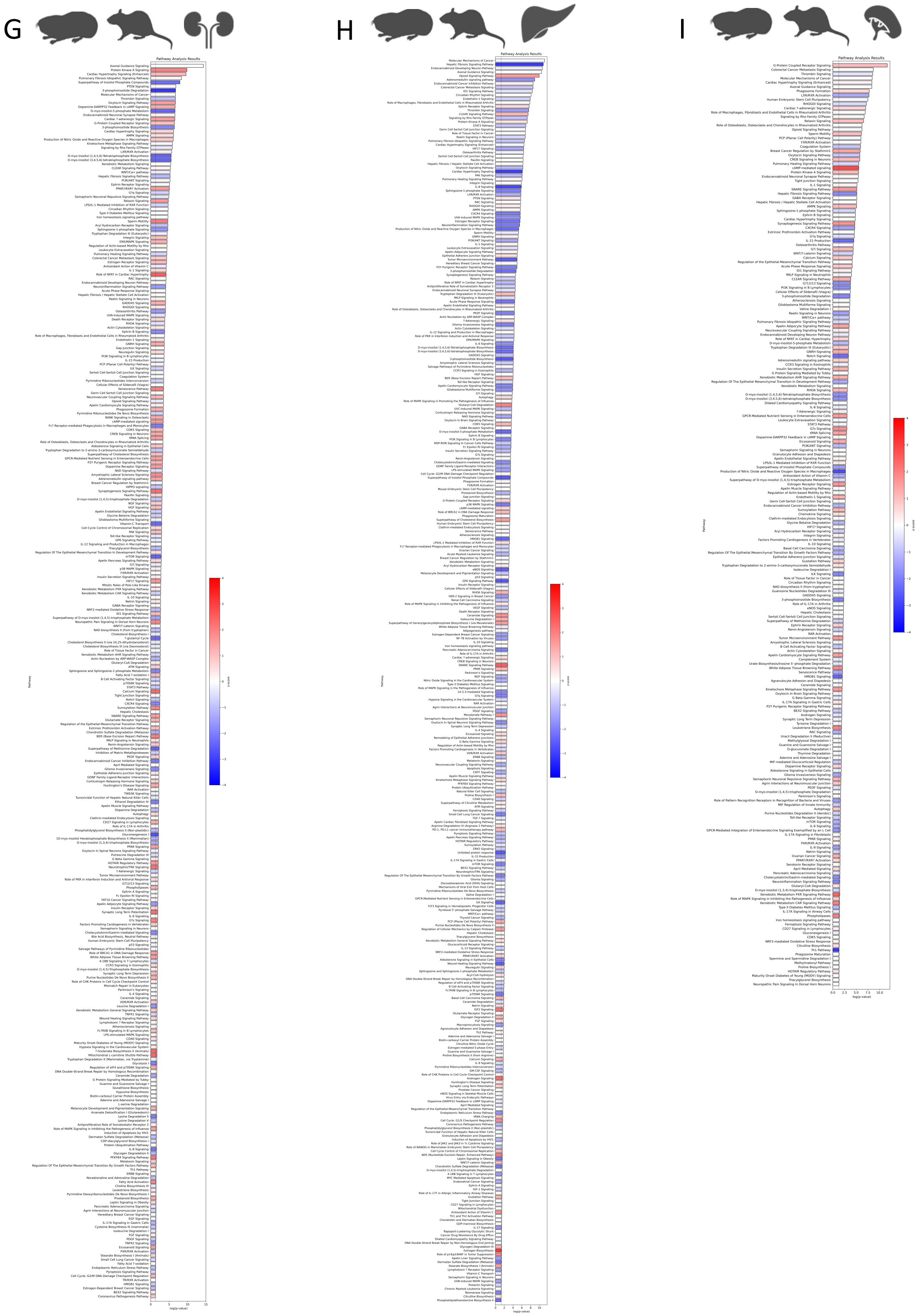

Bar plots of significantly enriched ($p < 0.05$) pathways among the differentially expressed genes. A-C: Hypoxia-regulated pathways in *Spalax* in kidney, liver, and spleen. D-F: Hypoxia-regulated pathways in rat in kidney, liver, and spleen. G-I: Enriched pathways in the interspecies comparison between *Spalax* and rat under normoxic conditions. The bar length indicates the p-value while the bar color indicates the z-score.
